# Aleurone supplementation enhances the metabolic benefits of training in Standardbred mares: impacts on glucose-insulin dynamics and gut microbiome composition

**DOI:** 10.3389/fphys.2025.1565005

**Published:** 2025-04-10

**Authors:** Berit Boshuizen, Lorie De Maré, Maarten Oosterlinck, Filip Van Immerseel, Venessa Eeckhaut, Constance De Meeus, Lindsey Devisscher, Carmen Vidal Moreno de Vega, Maarten Willems, Jean Eduardo De Oliveira, Guilherme Hosotani, Yannick Gansemans, Tim Meese, Filip Van Nieuwerburgh, Dieter Deforce, Katrien Vanderperren, Elisabeth-Lidwien Verdegaal, Cathérine Delesalle

**Affiliations:** ^1^ Department of Translational Physiology, Infectiology and Public Health, Research Group of Comparative Physiology, Faculty of Veterinary Medicine, Ghent University, Merelbeke, Belgium; ^2^ Equine Hospital Wolvega, Oldeholtpade, Netherlands; ^3^ Department of Large Animal Surgery, Anaesthesia and Orthopaedics, Faculty of Veterinary Medicine, Ghent University, Merelbeke, Belgium; ^4^ Department of Pathobiology, Pharmacology and Special Animals Faculty of Veterinary Medicine, Ghent University, Merelbeke, Belgium; ^5^ Gut-Liver ImmunoPharmacology Unit, Department of Basic and Applied Medical Sciences, Faculty of Medicine and Health Sciences, Ghent University, Ghent, Belgium; ^6^ Cargill Research and Development Centre Europe, Vilvoorde, Belgium; ^7^ Department of Pharmaceutics, Laboratory of Pharmaceutical Biotechnology, Ghent University, Ghent, Belgium; ^8^ Department of Morphology, Imaging, Orthopedics, Rehabilitation and Nutrition, Faculty of Veterinary Medicine, Ghent University, Merelbeke, Belgium; ^9^ Equine Health and Performance Centre, School of Animal and Veterinary Sciences, Roseworthy Campus, University of Adelaide, Adelaide, SA, Australia

**Keywords:** metabolism, exercise, nutrition, microbiome, intravenous and per oral glucose tolerance test, insulin sensitivity

## Abstract

**Introduction:**

Aleurone, derived from the bran layer of grains like wheat and barley, has demonstrated positive effects on energy metabolism in pigs, mice, and untrained horses, influencing glucose-insulin dynamics and gut microbiome composition. Training itself enhances insulin sensitivity in horses, similar to the improvements in performance capacity observed in human athletes. This study aimed to investigate whether aleurone supplementation provides additional benefits to training by modulating insulin metabolism and gut microbiota in Standardbred mares.

**Methods:**

Sixteen Standardbred mares (aged 3–5 years) participated in a cross-over study with two 8-week training periods separated by 8 weeks of detraining. Each horse received either 200 g/day aleurone supplementation or a control diet. Insulin metabolism was evaluated using oral (OGTT) and intravenous (FSIGTT) glucose tolerance tests, measuring parameters such as Maximum_glucose_, AUC_glucose_, Maximum_insulin_, AUC_insulin_, Time to peak_insulin_ (OGTT), Acute Insulin Response to Glucose (AIRg), glucose effectiveness (Sg), and disposition index (DI) (FSIGTT). Fecal samples underwent metagenomic analysis to assess alpha and beta diversity and microbial composition.

**Results:**

Training alone: Training significantly improved OGTT parameters by decreasing Maximum_insulin_ (*P* = 0.005) and AUC_insulin_ (*P* = 0.001), while increasing Time to peak_insulin_ (*P* = 0.03), indicating enhanced insulin sensitivity. FSIGTT results also showed a decrease in logAIRg (*P* = 0.044). Training with Aleurone: Aleurone supplementation further reduced FSIGTT AIRg (*P* = 0.030), logAIRg (*P* = 0.021) while increasing glucose effectiveness (Sg; *P* = 0.031). These findings suggest aleurone improves insulin sensitivity, glucose disposal, and fasting glucose regulation beyond training. Microbiome analysis revealed training decreased *Pseudomonas*, associated with dysbiosis, while aleurone reduced inflammation-associated *Desulfovibrio*. Beta diversity metrics showed no significant changes.

**Conclusion:**

Aleurone supplementation enhances training-induced improvements in glucose metabolism and fecal microbiota composition, which could offer potential benefits for equine athletes by optimizing metabolic flexibility. It also supports improvements in glucose and insulin dynamics, particularly by further enhancing insulin sensitivity and glucose-mediated disposal. Future studies should investigate the mechanisms of aleurone at the muscle and gut level and explore its potential applications for metabolic disorders such as Equine Metabolic Syndrome.

## Introduction

Aleurone is a specific layer within the bran fraction of wheat, barley, rye, and oat, that is deemed to be responsible for the beneficial health effects associated with the consumption of whole-grain products (Neyrinck et al., 2008; Rosa et al., 2014; Keaveney et al., 2015; Costabile et al., 2022; Fava et al., 2022). Previous research has demonstrated that aleurone supplementation enhances insulin sensitivity in non-trained horses, while also inducing significant shifts in gut microbiome composition and metabolic output (Boshuizen et al., 2021).

Insulin sensitivity is a critical aspect of the glucose metabolism and overall metabolic health in both animals and humans. In horses, insulin resistance is associated with an increased risk for developing conditions such as Equine Metabolic Syndrome (EMS) and laminitis (Asplin et al., 2007; Walsh et al., 2009; de Laat et al., 2015; Karikoski et al., 2015; Durham et al., 2019). An increase in insulin sensitivity will lead to improved energy generation, optimized glycogen storage, enhanced lipid utilization, reduced fatigue, promoted muscle recovery and growth, metabolic disorder prevention, and improved cardiovascular efficiency in both animals and humans (Duncan et al., 2003; Stewart-Hunt et al., 2006; Menzies-Gow et al., 2014). Routine sport horse feeding- and housing management, including meal feeding, influence insulin secretion (Piccione et al., 2013). Understanding the factors that modulate insulin sensitivity is therefore essential for improving health, wellbeing and performance in sports horses.

During an oral dosage trial, it was shown that feeding ≥200 g of aleurone to untrained horses significantly changes the glucose- and insulin response to a meal: time to peak of both blood glucose and insulin increased, while Area Under the Curve (AUC) of glucose and peak glucose remained the same and AUC_insulin_ and maximum insulin decreased. Until now, there are no studies available investigating the effect of aleurone supplementation on glucose and insulin dynamics in exercising/trained horses. The effect of exercise on equine metabolic physiology is complex and dynamic, holding effects not only on insulin sensitivity, but also on inflammatory and endocrine parameters (Assenza et al., 2018) and the gut microbiome (Cook et al., 2016; Janabi et al., 2016; 2017). Therefore, the metabolic effects of feed supplements such as aleurone should not be assumed the same for non-trained sedentary individuals as for exercising horses.

Numerous studies have explored the impact of exercise (both endurance and power exercise) and training on insulin sensitivity (Arfuso et al., 2019). Research by Lacombe et al. (2003), McCutcheon et al. (2002) and Stewart-Hunt et al. (2006) has shown that muscular GLUT four expression increases in horses in response to both acute exercise and prolonged training periods. However, in horses, other findings have challenged this narrative (Manso Filho et al., 2017, Filho et al., 2020; Nout et al., 2003; Pratt et al., 2007; Valberg et al., 2023; Vidal Moreno de Vega et al., 2023). The latter studies indicate that insulin-driven glucose transporters are either not influenced or downregulated following acute exercise, and even more so after 8 weeks of standardized training, suggesting that glucose might not be as pivotal a fuel source in trained horses, as previously thought. Alternative energy pathways including amino acid metabolism have been suggested to play an important role in equine muscular metabolism (Arfuso et al., 2019). The discrepancy is further supported by the prolonged period (48–72 h) required for horses to replenish muscular glycogen stores (Lacombe et al., 2004).

Given these contradictory findings, it becomes crucial to investigate the evolution of insulin sensitivity in horses undergoing training and aleurone supplementation. This study aimed to elucidate the role of exercise with and without aleurone supplementation in modulating insulin sensitivity to better understand the physiological mechanisms at play. By examining the separate and combined effects of training and aleurone supplementation on glucose and insulin dynamics and shifts in the fecal microbiome, we hope to contribute to a more comprehensive understanding of insulin regulation in horses and inform strategies for managing insulin dysregulation.

## Materials and methods

### Animals

The study involved a uniform group of sixteen adult client-owned Standardbred mares (3–5 years old) that were not involved in either training or competition during the 4 months prior to start of the study. Their average body mass was 453 kg ± 31 kg before training and 449 kg ± 36 kg after training; body condition score remained at 4.5 ± 0.5 (on a scale of one–9 (Henneke et al., 1983)) throughout the entire trial and assessed on a weekly basis. All horses were stabled at the same training facility in the same barn, housed in individual boxes (14 m^2^) on straw bedding with visual and tactile contact with their neighbours. The horses were turned out daily for 4 h in a sand paddock for social interaction and had *ad libitum* access to tap water and good quality hay (>1 kg hay/100 kg BW). During the entire study hay was fed from the same batch. The quality and composition of the hay batch was monitored visually and analyzed employing a Weende analysis throughout the study.

### Dietary composition and feeding

Horses were trained using a standardized protocol for two periods of 8 weeks (see below), with and without 200 g/d wheat aleurone supplementation (Boshuizen et al., 2021) (8 weeks detraining in between training periods) in a cross-over design. In the first training period eight of the 16 horses received feed with aleurone supplementation and the other eight horses received feed without aleurone supplementation. After the detraining period, in the second training period the groups were switched. A control concentrate pellet feed and an isocaloric aleurone (GrainWise™ Wheat Aleurone; Horizon Milling; Minneapolis, Minnesota, USA Cargill) (Bohm et al., 2003; Brouns et al., 2012) containing concentrate pellet feed were manufactured based upon calculations using a software program (*Horse Ration Calculator FND-2012*) applying settings to cover energy and dietary needs for the planned training program of adult Standardbred mares of 450 kg. Briefly, both diets had the same macronutrient composition, with the difference that in the non-aleurone diet, the aleurone component was replaced by wheat bran, which resulted in the same energy and digestible protein levels (Supplementary Table S1). Based upon these calculations, each horse was fed twice a day (8 a.m. and 8 p.m.), a total of 3 kg of concentrate feed per day.

### Training regime

**FIGURE 1:**
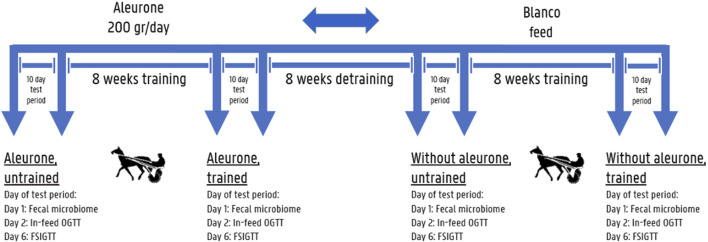
Cross-over study set-up: The first training period 8 mares started in the aleurone supplemented group and at the same time the other 8 mares started in the blanco group, the second training period these mares were all crossed-over into the other group. An in-feed Oral Glucose Tolerance Test (OGTT) (day 2/10) and a Frequently Sampled Intravenous Glucose Tolerance Test (FSIGTT) (day 6/10) were performed on all horses on respectively day 2 and d 6 (both resting days) before and after each 8 weeks training period. On day 1/10 before and after each training period fecal samples were collected for microbiome analysis.

All horses were harness trained 4 days a week on the same racetrack (Figure 1) for eight consecutive weeks in a cross-over model, with 8 weeks detraining in between by the same experienced driver. Horses were equipped with a GPS tracker and heart rate monitoring system (Polar® Equine H7, Polar Electro Oy, Finland). Throughout the training trial, the aerobic threshold of each horse was assessed by means of a specific Standardized Exercise Test (SET) using lactate minimum speed (LMS) testing before and after each 4 weeks of training, to adapt individual training speed to allow for optimal aerobic conditioning (De Maré et al., 2022). Each training session consisted of a warming-up period of 10 min jogging (±20 km/h), followed by either 30 min of aerobic training (3 days a week, ±25 km/h, mean HR ± 150 BPM) at aerobic speed (90% LMS) or interval training (day 4) consisting of 3 × 3 min at high speed at anaerobic 130% LMS speed (±35 km/h, mean HR ± 190 BPM) and trotted in between the 3 min high speed intervals at aerobic 90% LMS speed.

### Monitoring

The horses vital signs, including heart rate, temperature, respiratory rate, capillary refill time, appetite, and fecal production, were monitored twice daily. They were given 2 weeks to acclimate before the start of the training trial. Each horse was monitored daily by a veterinarian and remained in good health throughout the study, with no adverse events reported. For general health monitoring, venous blood samples were collected and analysed for a complete blood count and clinical biochemistry at the beginning and end of each training period. All blood parameters remained within the reference range for all horses throughout the entire trial.

### Experimental procedure

Before and after each training period sample collection for fecal microbiome analysis, in-feed oral glucose tolerance tests (OGTT) and frequently sampled glucose tolerance tests (FSIGTT) were executed during a 10-day testing period, according to the timeline presented in Figure 1. An in-feed OGTT (day 2/10) and FSIGTT (day 6/10) were performed on all horses on respectively day 2 and day 6 (both resting days) before and after each 8-week training period. On day 1/10, before and after each training period, fecal samples were collected for microbiome analysis. Horses subjected to these tests whilst assigned to the aleurone-supplemented group were labeled as “Aleurone, untrained” to assess the pre-training and pre-supplementation situation, and after 8 weeks of training in combination with aleurone supplementation they were labeled as “Aleurone, trained.” These two time points were compared. Horses in the non-supplemented group were labeled “Without aleurone, untrained” and after 8 weeks of training “Without aleurone, trained.” These two time points were compared as well. In addition, the “Aleurone, trained” group was compared with the “Without aleurone, trained” group to assess the effect of aleurone on top of training.

An *in feed OGTT* was executed as previously described (Jeffcott et al., 1986; Frank and Geor, 2014; Smith et al., 2016). Briefly, horses were fasted from 10 p.m. the day before and the test was performed between 8 a.m. and 2 p.m. on each occasion. After the collection of a venous basal blood sample on a plain and Na^+^ fluoride coated tube, a meal consisting of 0.5 g of carbohydrates/kg BW was administered. This meal was comparable in size, energy content and composition to the regular diet of the studied horses and encompassed 312 g of concentrate feed/100 kg BW. Blood samples were collected every 30 min for 5 h until blood glucose levels returned to baseline values. Serum tubes were centrifuged at 3.000 *g* at room temperature for 6 min and kept at 4°C until insulin analysis was performed within 24 h. Plasma glucose was immediately measured using the handheld Alphatrak® (Zoetis, Zaventem, Belgium) and serum insulin concentrations were measured using a chemiluminescent immunoassay (Immulite® 1,000 Immunoassay System, Siemens Healthcare Diagnostics, Inc., Tarrytown, NY) as previously described by Leschke et al. (2019). Plasma glucose concentrations are reported as mg/dL and serum insulin concentrations as mU/L. Post prandial glucose and insulin time profiles were constructed and following curve parameters were calculated for statistical analysis: Maximum_glucose_ (mg/dL), Time to peak_glucose_ (minutes), AUC_glucose_, ([mg/dL] x minutes) Maximum_insulin_ (mU/L), AUC_insulin_ ([mU/L] x minutes) which was divided into 0–210 min, 0–120 min and 0–60 min, Time to peak_insulin_ (minutes), AUC_glucose_/AUC_insulin_ and Basal_glucose_/Basal_insulin_. The AUCs were calculated using the trapezoidal method.

Before and after each training period a FSIGTT was performed on day 6 of the 10-day testing period. Horses were fasted overnight, and an intravenous catheter was placed in the jugular vein 1 h before collection of the basal blood sample. A bolus of 300 mg/kg BW glucose was administered within 1 min as a 40% glucose solution (Glucose 40%, Braun, Melsungen, Germany). Blood samples for both glucose and insulin measurements were collected in plain- and Na^+^ fluoride coated tubes after respectively 5, 15, 25, 35, 45, 60, 75, 90, 105, 120, 135, 150, 165, 180, 195, 210, 225 min. Serum tubes were centrifuged at 3.000 *g* at room temperature for 6 min and kept at 4°C until insulin analysis was performed. Plasma glucose was immediately measured using Alphatrak® (Zoetis, Zaventem, Belgium) and serum insulin concentrations were measured using a chemiluminescent immunoassay (Immulite® 1,000 Immunoassay System, Siemens Healthcare Diagnostics, Inc., Tarrytown, NY) as previously described by Leschke and colleagues (Leschke et al., 2019). Plasma glucose concentrations are reported as mg/dL and serum insulin concentrations as mU/L. A minimal model analysis was performed (MINMOD Millennium version 6.02) to determine glucose and insulin dynamics. Outcome variables included: acute insulin response to glucose (AIRg (mU/L/minute)) and logAIRg, insulin sensitivity (IS (L/minute/mU)), insulin mediated glucose disposal, glucose effectiveness (Sg (per minute)); glucose mediated glucose disposal and disposition index (DI (DI = IS x AIRg)); glucose effectiveness at zero insulin (GEZI) basal glucose (BG (mg/dL)) and basal insulin (BI (mU/L). (Boston et al., 2003). An overview of all involved MinMod parameters and their respective physiological connotation is provided in Table 1.

**TABLE 1:** An overview of the different calculated MinMod and FSIGTT parameters and their physiological connotation (Pacini and Bergman, 1986; Boston et al., 2003; Pritchard et al., 2019).

MinMod/FSIGTT parameter	Units	Abbreviation	Characteristics and/or physiological connotation
Acute Insulin Response to Glucose	mU/L/min	AIRg	• Measurement of the fast- and first response of the body to IV glucose administration
			• A lower AIRg corresponds with reduced insulin-resistance
			• Adequacy of insulin secretion
logAIRg	—	logAIRg	• Logarithmic transformation of AIRg
			• Significantly correlated to SI clamp
Insulin Sensitivity	L/min/mU	IS	• Insulin-mediated glucose disposal
			• Estimated tissue insulin sensitivity
Glucose effectiveness	per min	Sg	• Glucose-mediated glucose disposal
			• Ability of glucose *per se* to stimulate its own uptake and to suppress glucose production under basal insulin concentration
			• Capacity of cells to take up glucose at basal insulin
			• Glucose-driven glucose uptake
Disposition index	SI x AIRg	DI	• Measure of the ability of β -cells to secrete insulin
			• A measure of β -cell function and the ability of the body to dispose of a glucose load

### Microbiome analysis

Fecal samples were collected from the rectum before and after each training period, with or without aleurone supplementation on the first day of the 10-day testing period (Figure 1). The samples were immediately snap-frozen and stored at −80°C until further processing. DNA extraction was performed using the QIAamp Fast DNA Stool Mini Kit (Qiagen®). Briefly, 10 g of each fecal sample was homogenized in 90 mL PBS using a stomacher and filtered through a cell strainer (70 µm). Fecal pellets were washed in PBS twice before commencing DNA extraction using the manufacturers recommendations. DNA quantity and quality were assessed using a NanoDrop 1,000 spectrophotometer (Thermo Scientific). DNA quality was considered acceptable if the 260/280 ratio was between 1.8 and 2.0 and the 260/230 ratio was between 2 and 2.2. Within 24 h after DNA extractions were completed, PCR amplification of the V3-V4 region of the 16S rRNA gene was performed by using respectively a forward primer:

5′TCGTCGGCAGCGTCAGATGTGTATAAGAGACAGCCTACGGGNGGCWGCAG and 5′TCTCGTGGGCTCGGAGATGTGTATAAGAGACAGGACTACHVGGGTATCTAATCC as reverse primer. The first round of PCR amplification was performed in a total volume of 25 μL; containing 12.5 ng microbial genomic DNA, 12.5 μL of 2 × KAPA HiFi HotStart Ready Mix (Sigma-Aldrich, Overijse, Belgium) and 20 µm of each primer. Thermocycling conditions included an initial denaturation step at 95°C for 3 min, followed by 25 cycles of denaturation at 95°C for 30 s, annealing at 55°C for 30 s and extension at 72°C for 30 s, and a final extension cycle at 72°C for 5 min. The gene specific primers were appended with adapter sequences that are compatible with a subsequent index PCR that attaches dual indices and Illumina sequencing adapters using the Illumina Nextera XT Index Kit. This way, up to 96 libraries can be created and pooled together for sequencing. Libraries are quantified using qPCR with primers on the required Illumina library adaptor sequences (following the Illumina qPCR quantification protocol guide) and pooled equimolarly. The equimolar pool is denatured and diluted following Illumina protocols to produce a final 4.5 pM sequencing library. Twenty percent denatured Illumina PhiX Control V3 library was admixed to increase sequence diversity of this final library. Cluster generation and 2 × 300 paired-end sequencing is performed in one Illumina Miseq flowcell. Using Illumina Miseq 300-bp paired-end sequencing, paired-end reads are generated of which the ends are overlapped. The overlapping reads can be stitched to form high-quality, full-length sequences of the V3 and V4 region.

This study was approved by the Animal Ethics Committee of the Ghent University EC 2016/40.

### Statistical analysis

*For both the in-feed OGTT and the FSIGTT* descriptive statistics of continuous variables are expressed as mean ± SD and 95% CI. Statistical analysis was performed using R software (version 3.6) (Ihaka and Gentleman, 1996). Normal distribution of the data was assessed using the Shapiro-Wilk test with alpha level set to 0.05. A paired samples t-test was applied for normally distributed data, while for data presenting significant difference from normal distribution, a paired Wilcoxon test was used.

Data preparation and *metagenomics* analyses were all done using QIIME2 (v2019.4) (Caporaso et al., 2010; Zhang et al., 2014; Bolyen et al., 2019) unless otherwise mentioned. This includes sequenced read-pair quality trimming, merging into reconstructed amplicons, operational taxonomic unit (OTU) picking, taxonomic assignment and phylogenetic reconstruction. To build OTU tables and trees, open-reference OTU picking was performed against the Greengenes 16S reference collection (release 13.8) (DeSantis et al., 2006). To verify if sequencing was adequate and reached feature saturation, we visually inspected rarefaction plots depicting the number of uniquely identified features at increasing sequencing depth for all individual samples. Alpha diversity metrics Shannon’s Diversity, Faith’s Phylogenetic Diversity and observed OTUs were calculated for each sample group comparison and statistically analyzed using a pairwise Kruskal–Wallis test. Beta diversity metrics Weighted UniFrac, Unweighted UniFrac and Bray-Curtis were also calculated for each comparison of sample groups and statistically analyzed with a PERMANOVA test to assess the significance of the differences. To identify taxonomic features that were differentially abundant between conditions, an analysis of the composition of the microbiome was done in R using ANCOM (v2.1) (Mandal et al., 2015), including horse identity as a blocking factor in the design. According to the recommendations of the author’s software, the minimum threshold for significantly different taxa was set at 70% for the W statistic (W_0.7_). Fold change (FC) was calculated using the mean centered log ratio difference.

Prediction of shifts in *fecal microbiome metabolic output* due to either training or aleurone supplementation was conducted using the predictive metagenome analysis tool, Phylogenetic Investigation of Communities by Reconstruction of Unobserved States (PICRUSt) (Langille et al., 2013), with the Metacyc metabolic pathways database (Caspi et al., 2020). To identify differential pathways between conditions, the resulting pathway abundances were analyzed in STAMP (v2.1.3) (Parks et al., 2014) using a two-group Welch’s t-test.

### Correlation between FSIGTT and microbiome parameters spearman

Correlation analysis was performed to examine the relationship between changed indices derived from the FSIGTT and differences in relative abundance of bacterial genera from the fecal microbiome caused by either feeding aleurone and/or training. Significance was set at *P* ≤ 0.05.

## Results

### General health monitoring

Besides daily vet checks, venous blood samples were analyzed for complete blood count and clinical biochemistry at the beginning and end of each training period. All measured parameters remained within the reference range throughout the entire study.

### In-feed OGTT

Only training had a significant effect on distinct OGTT parameters, no additional modulation by aleurone supplementation could be detected (Table 2). Training without aleurone induced significant OGTT curve parameter changes. OGTT Maximum_insulin_ was significantly lower (*P* = 0.005), Time to peak_insulin_ was significantly higher (*P* = 0.034) and AUC_insulin 0–210_ was significantly lower for “without aleurone, trained” compared to “without aleurone, untrained” (*P* = 0.001). AUC_insulin 0–120_ (*P* = 0.030) was also significantly lower after training, however, AUC_insulin 0–60_ was not (*P* = 0.229). AUC_glucose_/AUC_insuline_ was significantly higher after training (*P* = 0.010). Maximum_glucose_ (*P* = 0.207), Time to peak_glucose_ (*P* = 0.769), and AUC_glucose_ (*P* = 0.083) did not change significantly after training without aleurone.

**TABLE 2:** Mean and 95% Confidence Intervals of measured and calculated indices from the in-feed OGTT in sixteen Standardbred mares before and after training; and with and without aleurone supplementation. Significant differences (*P* < 0.05) are indicated in bold. AUC_glucose_ = Area under the curve glucose concentration time-curve. AUC_insulin_ = Area under the curve insulin concentration time-curve.

	Effect of training without aleurone supplementation					Effect of training with aleurone supplementation					Effect of aleurone supplementation on top of training
	Without aleurone, untrained		Without aleurone, trained			Aleurone, untrained		Aleurone, trained			Without aleurone, trained vs aleurone, trained
Variable	Mean ± SD	95% Cl	Mean ± SD	95% Cl	*P* Value	Mean ± SD	95% Cl	Mean ± SD	95% Cl	*P* Value	*P* Value
Maximum_glucose_	8.3 ± 1.0	7.8–8.8	8.7 ± 1.5	7.9–9.4	0.207	8.5 ± 1.5	7.4–9.2	8.3 ± 1.4	7.6–8.9	0.588	0.319
Time to peak_glucose_	102.0 ± 40.6	81.5–122.5	106.0 ± 27.5	92.1–119.9	0.769	108.8 ± 36.1	91.0–126.5	105.0 ± 29.0	90.8–119.2	0.763	1.000
AUC_glucose_	1,504.7 ± 156.2	1,425.7–1,583.7	1,560.8 ± 213.5	1,452.7–1,668.9	0.083	1,526.0 ± 189.8	1,433.0–1,619.1	1,501.6 ± 219.7	1,393.9–1,609.2	0.622	0.284
Maximum_insulin_	69.3 ± 66.2	35.8–102.7	36.4 ± 28.5	22.0–50.8	**0.005**	43.4 ± 28.3	29.5–57.3	72.4 ± 84.5	31.0–113.8	0.193	0.762
Time to peak_insulin_	140 ± 52.8	113.3–166.7	168 ± 33.6	151.0–185.0	**0.034**	120.0 ± 42.4	99.2–140.8	146.3 ± 45.0	124.2–168.3	0.089	0.195
AUC_insulin 0–60_	944.5 ± 652.4	614.3–1,274.7	704 ± 523.0	439.3–968.7	0.229	794.4 ± 553.3	523.3–1,065.5	1,243.7 ± 1,450.3	533.1–1954.3	0.123	0.259
AUC_insulin 0–120_	3,607.3 ± 3,324.6	1924.8–5,289.8	2,184.5 ± 1,640.6	1,354.2–3,014.8	**0.030**	2,601.1 ± 1723.2	1756.7–3,445.5	4,215.8 ± 5,587.8	1,394.0–1,609.2	0.231	0.561
AUC_insulin 0–210_	8,474.4 ± 7,110.4	4,876.1–12072.7	4,237.4 ± 3,037.0	2,700.5–5,774.3	**0.001**	5,562.6 ± 3,781.1	3,709.8–7,415.4	9,143.8 ± 11,278.9	3,617.2–14670.5	0.144	0.978
AUC_glucose_/AUC_insulin_	0.326 ± 0.253	0.198–0.453	0.554 ± 0.374	0.365–0.743	**0.010**	0.445 ± 0.324	0.286–0.603	0.423 ± 0.460	0.197–0.648	0.348	0.720
Basal_glucose_/Basal_insulin_	79.3 ± 29.0	64.6–94.0	78.9 ± 26.5	65.5–92.3	0.977	73.5 ± 33.3	57.2–89.8	83.3 ± 28.8	69.2–97.4	0.214	0.176

There was no additional effect of aleurone supplementation on top of training on OGTT parameters. Training with aleurone supplementation (“aleurone, trained”) showed a trend, though not significant, towards a delayed OGTT Time to peak_insulin_ (*P* = 0.089) when compared to the starting values (“aleurone, untrained”).

### FSIGTT

Aleurone induced significant additional effects on FSIGTT variables on top of training (Table 3). Training without aleurone decreased the logAIRg (*P* = 0.044) but had no other significant effects on glucose dynamics (Table 3).

**TABLE 3:** Mean and 95% Confidence Intervals of measured and calculated indices from the FSIGTT in sixteen Standardbred mares before and after training; and with and without aleurone supplementation. Significant differences (*P* < 0.05) are indicated in bold. AIRg = acute insulin response to glucose; DI = IS x AIRg or disposition index; IS = insulin sensitivity; Sg = glucose effectiveness or glucose mediated glucose disposal.

	Effect of training without aleurone supplementation					Effect of training with aleurone supplementation					Effect of aleurone supplementation on top of training
	Without aleurone, untrained		Without aleurone, trained			Aleurone, untrained		Aleurone, trained			Without aleurone, trained vs aleurone, trained
Variable	Mean ± SD	95% Cl	Mean ± SD	95% Cl	*P* Value	Mean ± SD	95% Cl	Mean ± SD	95% Cl	*P* Value	*P* Value
AIRg	148.1 ± 119.2	74.2–221.9	3.8 ± 97.3	33.5–154.1	0.172	115.0 ± 124.1	47.5–182.5	62.5 ± 64.8	27.3–97.7	**0.030**	**0.004**
logAIRg	4.699 ± 0.840	4.179–5.219	3.893 ± 1.433	3.004–4.781	**0.044**	4.365 ± 0.860	3.897–4.832	3.717 ± 1.093	3.12–4.31	**0.021**	**0.010**
DI	26,390 ± 53,385	−6,698–59479	27,427 ± 47,626	−2091–56947	0.771	35,816 ± 42,344	12,797–58834	29,221 ± 26,214	14,971–43471	0.409	0.468
IS	126.4 ± 181.1	14.1–238.7	624.8 ± 684.1	200.8–1,048.8	0.131	641.2 ± 917.0	142.8–1,139.7	538.0 ± 476.9	278.7–797.2	0.641	0.578
Sg	0.0123 ± 0.005	0.009–0.015	0.011 ± 0.003	0.009–0.012	0.340	0.0120 ± 0.004	0.0097–0.0142	0.0084 ± 0.0032	0.0067–0.0102	**0.031**	0.161

Training with aleurone (aleurone, untrained vs aleurone, trained) significantly decreased AIRg (*P* = 0.030), logAIRg (*P* = 0.021), and Sg, also known as glucose mediated glucose disposal (*P* = 0.031).

When comparing the FSIGTT variables after training with aleurone (“aleurone, trained”) to those after training without aleurone (“without aleurone, trained”), AIRg and logAIRg were significantly lower in the aleurone group (*P* = 0.004 and *P* = 0.010 respectively).

### Metagenomics

Raw sequencing reads were quality-trimmed, denoised and assembled into amplicon sequences, resulting in final amplicon counts ranging from 17,162 to 262,524. This corresponds with a recovery rate of 23%–38% of the reads, except for sample 30 (from the “aleurone, trained” group) for which only 8% was recovered. Features showing a frequency below 86 (0.1% of the mean frequency per sample) or which were not present in a total of seven samples (half size of the smallest group) were removed from the analysis. Filtered amplicons were assigned an operational taxonomic unit (OTU) based on their sequence and were linked to known microorganisms by comparison to the Greengenes 16S collection (release 13.8). The rarefaction plot showed that the saturation for observed OTUs was satisfactory after sampling 18,000 amplicons.

#### Diversity metrics

The following diversity metrics were calculated using a sampling depth of 18.298, which removed one sample (sample 30) that was part of the ‘aleurone, trained’ group. The microbiota richness and alpha diversity based on Shannon’s diversity, Faith’s Phylogenetic Diversity and observed OTUs were similar before and after aleurone supplementation. The weighted UniFrac distance followed by PCoA and the Bray-Curtis distance showed no distinct clustering between samples taken before and after 8 weeks of supplementation.

##### Alpha diversity

Alpha diversity was defined by respectively Shannon’s diversity (a quantitative measure of community richness), Faith’s Phylogenetic Diversity (a quantitative measure) and observed OTU’s (a qualitative measure). There were no significant differences in Shannon’s, Faith’s Phylogenetic Diversity and observed OTU’s measure of diversity between ‘without aleurone, untrained’ and ‘without aleurone, trained’. There was also no effect of adding aleurone to the training on these metrics (comparing ‘Aleurone, untrained’ with ‘Aleurone, trained’) nor when the results of these measures of alpha diversity were compared between ‘aleurone, trained’ with ‘without aleurone, trained’.

##### Beta diversity

Beta diversity was defined by (1) unweighted UniFrac distance, (a qualitative measure of community dissimilarity that incorporates phylogenetic relationships between the features), (2) quantitative measures of the weighted UniFrac- and (3) Bray Curtis distance. For all three measures no effect of training was found when comparing ‘without aleurone, untrained’ with ‘without aleurone, trained’.

A PERMANOVA group analysis using the Weighted UniFrac- and Bray-Curtis distance metrics also revealed that the “aleurone, untrained” and “aleurone, trained” groups showed no statistical difference, also no significant difference was observed for the unweighted UniFrac.

When comparing the communities of “aleurone, trained” with “without aleurone, trained” no significant changes in beta diversity were found.

#### Differentially Abundant Features

Training alone induced a significantly decreased abundancy at the genus level of *Pseudomonas,* shown by ANCOM (W = 16). Pseudomonadaceae was also significantly decreased at the family level (W = 16). The Likelihood Ratio Test in EdgeR reported one significantly decreased genus: *Streptococcus* (logFC = −2.43, FDR = 0.032).

Both ANCOM and EdgeR did not reveal any differentially abundant features across the “aleurone, before training” and “aleurone, after training” groups at species level, genus level or family level (Supplementary Figures S1 and S2).

**FIGURE 2:**
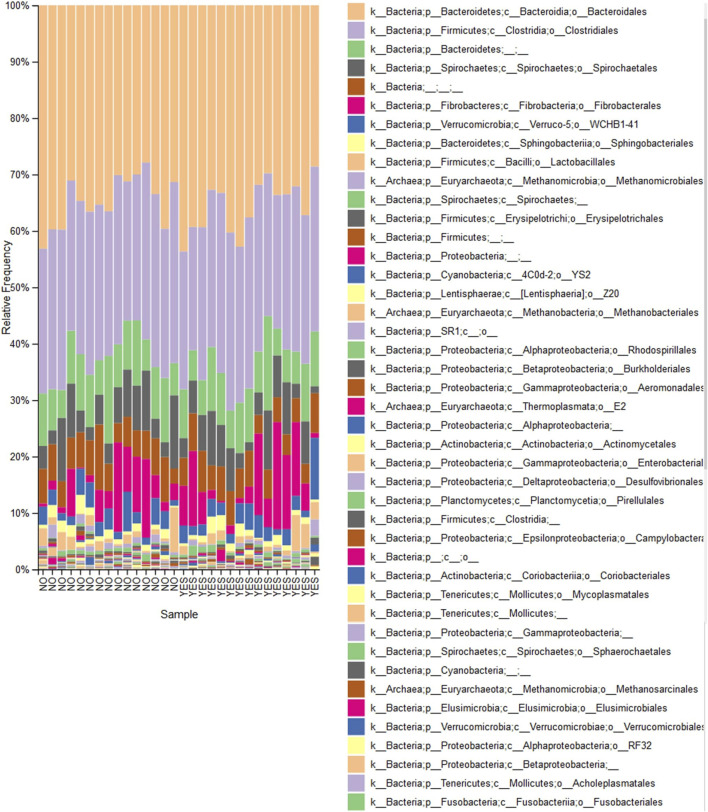
Taxa bar plot: Percentages of total identifiable reads for prominent bacterial orders identified following sequencing of fecal bacterial DNA. Each bar represents one sample either “Aleurone, trained” (YES) (n = 15) or “Without aleurone, trained” (NO) (n = 15). The key notes the dominant bacterial orders represented by each colored bar.

However, both ANCOM and EdgeR showed a decreased abundancy at genus level of the bacterial genera *Desulfovibrio* (W = 50) when comparing trained horses supplemented with aleurone to horses trained without aleurone supplementation (logFC = −4.356; FDR = 0.002) (Figure 2). This decreased abundancy was also shown at the family level of *Desulfovibrionace* by ANCOM (W = 40).

#### Metabolic microbial pathway up- and downregulation

##### Effect of training on metabolic microbial pathways

Training without aleurone supplementation changed relative pathway abundancy by downregulating glucose degradation pathways (2.66 fold change; *P* = 0.013), TCA cycle pathway (3.10 fold change; *P* = 0.011) and vitamin B12 biosynthesis at two levels in late cobalt incorporation (2.27 fold change and 1.94; *P* = 0.0002 and 0.011) (Table 4). Several aromatic compound degradation pathways were also significantly downregulated: 4-meyhylcatechol degradation (ortho-cleavage) was downregulated almost 5 times after training (4.87 fold change; *P* = 0.029) and the toluene degradation III pathway was downregulated 2 times (2.06 fold change 2.06; *P* = 0.003).

**TABLE 4:** Relative pathway abundancy when comparing the MetaCyc data from feces collected in week 0 and week 8 in the non-supplemented groups (“without aleurone, untrained” (n = 15) and “without aleurone, trained” (n = 15)), showing the effect of training on the pathway enrichment data of horses. The table below reports pathways with an uncorrected *P* value ≤0.05 and a fold change of <0.67 and >1.5. A fold change larger than one means the pathway was enriched in the “without aleurone, untrained” group while a fold change less than one means the pathway was enriched in the “without aleurone, trained” group.

Metabolic pathway description	Fold change	After training: mean rel. freq. (%)	After training: sd. (%)	Before training: mean rel. freq. (%)	Before training sd. (%)	*P* Value
superpathway of glycolysis, pyruvate dehydrogenase, TCA, and glyoxylate bypass	2.6604	0.0075	0.0037	0.0175	0.0199	0.0132
TCA cycle IV (2-oxoglutarate decarboxylase)	3.0977	0.011	0.007	0.0287	0.0341	0.0113
adenosylcobalamin biosynthesis II (late cobalt incorporation)	2.2731	0.0005	0.0004	0.0014	0.001	0.0002
toluene degradation III (aerobic) (*via* p-cresol)	2.0553	0.0001	0.0002	0.0003	0.0003	0.003
4-methylcatechol degradation (ortho cleavage)	4.8669	0.0002	0.0002	0.0005	0.0008	0.0292
cob (II)yrinate a,c-diamide biosynthesis II (late cobalt incorporation)	1.9428	0.0003	0.0003	0.0008	0.0007	0.0011
phenylacetate degradation I (aerobic)	4.0286	0.0003	0.0005	0.0009	0.0013	0.0451
superpathway of glyoxylate bypass and TCA	2.9052	0.0038	0.0019	0.0092	0.011	0.0147

##### Effect of aleurone supplementation combined with training on metabolic microbial pathways

All significantly changed pathways were downregulated after aleurone supplementation combined with training (‘aleurone, before training’ *versus* ‘aleurone, after training’) (Table 5). Glycolysis was downregulated for the archaeon family of *Pyrococcus spp*. (3.17 fold change; *P* = 0.029). Vitamin B12 biosynthesis was downregulated at two levels in late cobalt incorporation (2.10 and 2.13 fold change; *P* = 0.042 and *P* = 0.039). The superpathway of taurine degradation was downregulated significantly (2.23 fold change; *P* = 0.001). Aromatic compound toluene degradation was significantly downregulated at multiple levels including the superpathway of aerobic toluene degradation (2.42 fold change, 2.42 and 2.65; *P* = 0.046, *P* = 0.046 and *P* = 0.022). Training in combination with aleurone supplementation downregulated the methylaspartate cycle more than 2-fold (2.33 fold change; *P* = 0.044), as well as the superpathway of methane production by methanogens from the Archaea domain (3.18 fold change; *P* = 0.027). The biosynthesis of ergothioenine, a betaine related histidine, was also downregulated after training with aleurone (3.04 fold change; *P* = 0.039).

**TABLE 5:** Relative pathway abundancy when comparing the MetaCyc data from feces collected from the “aleurone, untrained” group (n = 15) with the “aleurone, trained” group (n = 15) showing the combined effect of training and oral aleurone supplementation to horses. The table below reports pathways with an uncorrected *P* value ≤0.05 and a fold change of <0.67 and >1.5. A fold change larger than one means the pathway was enriched in the “aleurone, untrained” group while a fold change less than one means the pathway was enriched in the “aleurone, trained” group.

Metabolic pathway description	Fold change	Aleurone, trained: mean rel. freq. (%)	Aleurone, trained: sd. (%)	Aleurone, untrained: mean rel. freq. (%)	Aleurone, untrained sd. (%)	*P* Value
Glycolysis V (Pyrococcus)	3.1669	0.0004	0.0004	0.0012	0.0018	0.0294
Adenosylcobalamin biosynthesis II (late cobalt incorporation)	2.1007	0.0006	0.001	0.0013	0.001	0.0418
Superpathway of taurine degradation	2.2268	0.0007	0.0007	0.0016	0.0008	0.0014
Toluene degradation I (aerobic) (via o-cresol)	2.4153	0.0015	0.0017	0.0036	0.005	0.0458
Toluene degradation II (aerobic) (via 4-methylcatechol)	2.4153	0.0015	0.0017	0.0036	0.005	0.0458
Superpathway of aerobic toluene degradation	2.6518	0.0001	0.0001	0.0001	0.0001	0.0215
Methylaspartate cycle	2.3256	0.0013	0.0017	0.003	0.0038	0.0439
Superpathway of methanogenesis	3.1772	0.0005	0.0006	0.0017	0.0025	0.0273
Ergothioneine biosynthesis I (bacteria)	3.0365	0.0001	0.0002	0.0004	0.0007	0.0299
Cob(II)yrinate a,c-diamide biosynthesis II (late cobalt incorporation)	2.1286	0.0004	0.0006	0.0008	0.0006	0.0393

##### Effect of aleurone supplementation on top of training on metabolic microbial pathway up- and downregulation

When comparing the predicted alterations in metabolic pathway expression of the fecal microbiome after 8 weeks of training with aleurone (‘aleurone, trained’) with 8 weeks of training without aleurone (‘without aleurone, trained’), multiple pathways were significantly changed by aleurone on top of training (Table 6): the aromatic biogenic amine degradation pathway was significantly increased with a fold change of 1.58 (*P* = 0.048). The following pathways were downregulated when aleurone was added to the training regime: the superpathway of glycol metabolism and degradation (0.32 fold change; *P* = 0.002), superpathway of taurine degradation (0.34 fold change; *P* = 0.002) and the photorespiration (0.51 fold change; *P* = 0.006).

**TABLE 6:** Relative pathway abundancy when comparing the MetaCyc data from feces collected after 8 weeks of training without aleurone (n = 15) vs. 8 weeks of training with aleurone (n = 15), to show the effect of oral aleurone supplementation on top of the effect of training. The table below reports pathways with an uncorrected *P* value ≤0.05 and a fold change of <0.67 and >1.5. A fold change larger than one means the pathway was enriched in the “aleurone, trained” group while a fold change less than one means the pathway was enriched in the “without aleurone, trained” group.

Metabolic pathway description	Fold change	Without aleurone, trained: mean rel. freq. (%)	Without aleurone, trained: sd. (%)	Aleurone, trained: mean rel. freq. (%)	Aleurone, trained sd. (%)	*P* Value
Superpathway of glycol metabolism and degradation	0.3226	0.0032	0.002	0.001	0.0009	0.0018
Superpathway of taurine degradation	0.3402	0.0015	0.0008	0.0005	0.0006	0.0017
Photorespiration	0.5063	0.0185	0.0072	0.0094	0.009	0.0064
Aromatic biogenic amine degradation (bacteria)	1.5763	0.0013	0.0007	0.002	0.0012	0.0484

#### Correlation between FSIGTT and fecalmicrobiome parameters

##### Correlation between FSIGTT and fecal microbiome parameters after training without aleurone supplementation

Training without aleurone decreased the logAIRg (*P* = 0.04) but had no other significant effects on glucose dynamics. However, differences in AIRg induced by training without aleurone supplementation were positively correlated to the relative abundance of the Peptostreptococcaceae family, but the correlation was less pronounced (R = 0.723, *P* = 0.003) compared to that found for training with an aleurone-supplemented diet (See Supplementary Table S1 for all significant correlations induced by training, the associated Spearman correlation coefficients and *P*-values).

Training without aleurone supplementation also showed a negative correlation between insulin sensitivity (IS) and the relative abundance of Peptostreptococcaceae and other families of the *Clostridiales. BF311* of the *Bacteroidetes* phyla was positively associated with IS, which means that the higher the IS the more abundant *BF311* was found. The strongest correlation found after training without aleurone supplementation of IS was a positive correlation with *Gammaproteobacteria* (R = 0.718, *P* = 0.004). In addition to this, a strong negative correlation of IS with the genera *Streptococcus* in the fecal microbiota was found (R = −0.664, *P* = 0.009) (Supplementary Table S2).

Differences in glucose effectiveness (Sg) induced by training alone showed a moderate negative correlation with the relative abundance of the bacterial family Oxalobacteraceae and a moderate negative correlation with the genus *Dorea* from the Lachnospiraceae family (Supplementary Table S2).

Differences in disposition index (DI) induced by training without aleurone supplementation showed a moderate negative correlation with the relative abundance of the genera *Clostridium* and Bacteroidaceae*.* The changes in abundance of the genera *Selenomonas, Spirochaeta* and the family of Victivallaceae are positively correlated with the differences in disposition index triggered by training (Supplementary Table S2).

##### Correlation between FSIGTT and fecal microbiome parameters after training with aleurone supplementation

**FIGURE 3:**
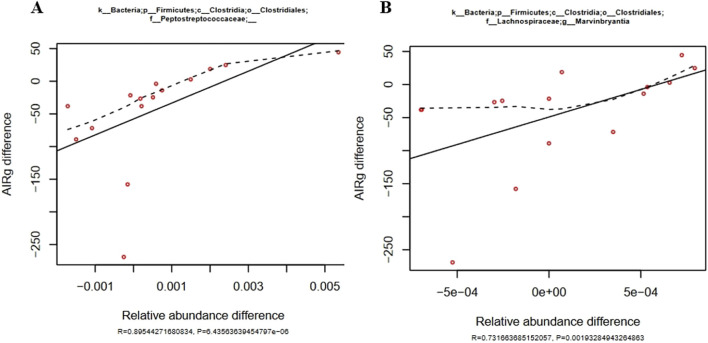
**(A)** Positive correlation of changes in Peptostreptococcaceae in the gut microbiome relative abundance (shown on the x-axis) with changes in AIRg (shown on the y-axis) induced by training and aleurone supplementation (n = 15). The solid line was fitted using the least squares method and is characterized by the R and P values. The dashed line represents a fitted spline curve. Below the figure the R- and P-value are shown as well. **(B)** Positive correlation of changes in *Marvinbryantia* in the gut microbiome relative abundance (shown on the x-axis) with changes in AIRg (shown on the y-axis) induced by training and aleurone supplementation (n = 15). Below the figure the R- and P-value are shown as well.

Relative abundance changes of eight different bacterial genera were significantly correlated to Acute Insulin Response to glucose (AIRg) changes that were induced by feeding aleurone and simultaneously training horses for 8 weeks. A low AIRg is associated with good metabolic health in humans and horses (García-Estévez et al., 2003; Slentz et al., 2009; Pratt-Phillips et al., 2015). Changes in AIRg after training with aleurone were significantly correlated to changes in abundance of the fecal microbiome. A positive correlation was shown for seven bacterial genera, and a negative correlation was found for one *Paraprevotellaceae* family. Two genera of the *Firmicutes Clostridia Clostridialis* family*,* namely, the Peptostreptococcaceae (R = 0.895, *P* < 0.001) and the Lachnospiraceae genus *Marvinbryantia* (R = 0.732, *P* = 0.002) (Figure 3) showed a strong and significant positive correlation with the changes in AIRg caused by aleurone supplementation in combination with training, whereas *Bacteriodetes* family *Paraprevotellaceae* was negatively correlated to AIRg. This translates into high abundance of the *Paraprevotellaeceae* family and low levels of Peptostreptococcaceae and *Marvinbryantia* with low levels of AIRg, in the more metabolic healthy horses.

**FIGURE 4:**
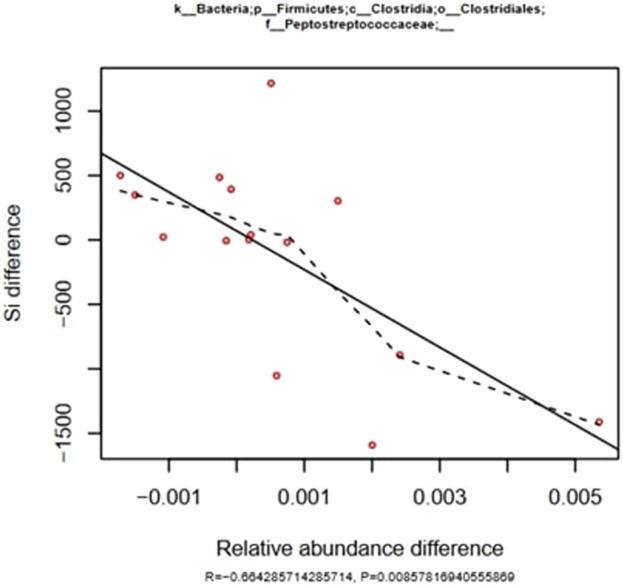
Effect of training and aleurone supplementation: correlation of differences in IS during a FSIGTT with differences in relative abundance of *Peptrostreptococcaceae* families in the fecal microbiome (n = 15). The solid line was fitted using the least squares method and is characterized by the R and P values. The dashed line represents a fitted spline curve.

Insulin sensitivity (IS) was not significantly modified by either training and/or feeding aleurone as shown by the minimal model analysis of the FSIGTT results, there were significant correlations between the changes in IS and the aleurone induced changes in relative abundance of six different bacterial genera (Supplementary Table S2). The correlations with IS are weaker when compared to the correlations with AIRg, however they were significant. Again, both *Peptostreptococcae* and *Marvobryantia* were correlated to a MINMOD parameter but in this case they were negatively correlated to IS differences, as could be expected because of the positive correlation with AIRg (Figure 4). This translates into higher gut microbiome relative abundance of *Peptostreptococcae* and *Marvobryantia* in horses with lower insulin sensitivity and therefore reduced metabolic health. The family of Ruminococcaceae and the genus of *Victivallis* are positively correlated with IS.

Differences in glucose effectiveness (Sg) induced by training and aleurone supplementation showed a strong negative correlation with the relative abundance of the bacterial family Moraxellaceae*,* a moderate negative correlation with *Anaeribiosprillum, F16,* the genus *Melissococcus,* the family of Veillonellaceae and Enterobacteriaceae and unidentified members of the *Spirochaetes* class. A positive correlation was found for *Paraprevotellaceae* and unidentified members of the *Clostridia* class and for the genus *Weisella.* This translates to adding aleurone to the diet of trained horses increased the capacity of cells to take up glucose at basal insulin levels and this is strongly correlated to a decreased abundance of Moraxellaceae families.

Differences in disposition index (DI) induced by training and aleurone supplementation showed a strong positive correlation with the changed relative abundance of the *Ruminococcus* genus. Differences in abundance of ten other bacterial genera were significantly correlated to differences in DI. Disposition index (DI) is a measure for β-cell function, measuring the ability of these islet cells to secrete insulin. The higher the DI the more insulin the body uses to transfer glucose into the tissues. When DI decreases in humans this is a sign of progressing from insulin resistance into β -cell depletion thus into diabetes mellitus type 2.

Differences in glucose effectiveness at zero insulin (GEZI) induced by training and aleurone supplementation were positively correlated to changes in relative abundance of the genera of *Peptostreptococcaecaea, Weisella,* Oxalobacteraceae*, Marvinbryantia* and the family *of* Lachnospiraceae. Only the *Victivallis* genera was negatively correlated to the GEZI differences.

## Discussion

This study investigated the effects of an 8 weeks training program with and without aleurone supplementation on glucose-insulin dynamics and fecal microbiome composition and it’s metabolic output in Standardbred mares. The findings highlight the complementary effects of aleurone supplementation in improving metabolic health and enhancing the effects of training. The primary outcomes assessed include various parameters from OGTT and FSIGTT, alongside comprehensive metagenomic analyses of fecal samples. An interesting finding in the current study was the difference in outcomes between the OGTT and FSIGTT parameters, with regard to training and aleurone supplementation effects. Aleurone supplementation has a more pronounced effect on FSIGTT parameters compared with OGTT parameters, where training has a predominant effect.

### Glucose and insulin dynamics

Our findings show that training alone significantly altered certain OGTT parameters, specifically decreasing OGTT Maximum_insulin_ and AUC_insulin_, while increasing Time to peak_insulin_. This suggests an improvement in insulin sensitivity as a result of training, consistent with previous studies demonstrating enhanced insulin dynamics following exercise training in horses and other species (Borghouts et al., 1999; Stewart-Hunt et al., 2006). Training without aleurone also reduced log AIRg in the FSIGTT, indicating a decreased acute insulin response to glucose, a marker associated with improved metabolic health (García-Estévez et al., 2003).

The physiological interpretation of the findings from the OGTT in this study, where training had a significant effect on several insulin-related parameters but no additional effect from aleurone supplementation on the OGTT test results, is somewhat surprising. In the current study, the effects of aleurone were predominantly exposed by the FSIGTT test results.

As expected, training also significantly influenced FSIGTT test results. Training without aleurone decreased logAIRg, which represents the logarithmic transformation of the AIRg. This parameter measures how much insulin is secreted in response to a glucose challenge. The decrease in logAIRg suggests that training alone leads to a reduced acute insulin response. This indicates that the training protocol applied in the current study improves insulin sensitivity, so the body needs less insulin to manage blood glucose levels. Likewise, training had a positive effect on several OGTT parameters.

There are several possible explanations as to why training predominantly affects OGTT parameters: Training improves glucose metabolism by enhancing muscle insulin sensitivity, and glycogen storage capacity. In OGTT, these training-induced adaptations lead to more efficient glucose uptake and utilization, reducing postprandial glucose excursions. Moreover, training has been shown to enhance incretin responses (e.g., GLP-1 secretion), which improves insulin release and glucose clearance after oral glucose intake. Probably, because OGTT depends more on these postprandial metabolic processes, the beneficial effects of training are more visible here compared to the FSIGTT. Aleurone on its turn had a greater effect on FSIGTT parameters. Also for this finding there are several different possible explanations: Aleurone is known to be rich in bioactive polyphenols, which can enhance peripheral insulin sensitivity by improving muscle and adipose tissue glucose uptake. These effects are more pronounced in FSIGTT, where glucose uptake into peripheral tissues (muscle, adipose) plays a dominant role, whereas in OGTT, gastrointestinal and hepatic mechanisms play a larger role in glucose clearance. Additionally, aleurone’s antioxidant and anti-inflammatory properties may improve insulin signalling at the tissue level, which is more directly reflected in FSIGTT-derived insulin sensitivity indices. The significant reduction in AIRg and logAIRg with training in combination with aleurone supplementation suggests a synergistic improvement in insulin efficiency. Most probably, aleurone enhances insulin signalling and glucose uptake in muscle tissue, especially when combined with exercise-induced metabolic adaptations.

These effects are particularly relevant for equine athletes, where efficient glucose utilization could contribute to enhanced performance capacity, especially in situations where acute strategic speed accelerations need to be realized after already executing long term relative high speed exercise. In this way, the so-called versatile winning fuel (glucose/glycogen) upon which such short bouts of finalizing accelerations can be realized by the competing horse, is effectively optimized due to the impact of training in combination with aleurone supplementation, enhancing both energy availability and metabolic efficiency during high-intensity performance.

The absence of effect of aleurone supplementation on OGTT results in the current study is in contradiction with those reported in a previous aleurone dosing trial performed in untrained horses (Boshuizen et al., 2021). In that study, feeding >200 g of aleurone to untrained horses significantly changed the glucose- and insulin response to a meal (OGTT): time to peak of both blood glucose and insulin increased, while AUC_glucose_ and peak glucose remained the same, and AUC_insulin_ and Maximum_insulin_ decreased. The different findings could be attributed to the fact that the effects of training overshadow the effects of aleurone supplementation in the current study.

In future studies, it would be valuable to explore the effects of aleurone beyond carbohydrate metabolism, investigating its potential impact on other metabolic pathways such as lipid metabolism, protein turnover, and mitochondrial function. Given the bioactive properties of aleurone, it may influence metabolic flexibility, inflammatory responses, or oxidative stress regulation, which could provide further insight into its broader physiological benefits. There is an increasing amount of evidence to support the view that insulin in horses plays a much more complex role than solely having the glucose metabolism as its main focus (Vidal Moreno de Vega et al., 2023). Previous research focused on evolution of glucose transporter expression (GLUT4, 8, and 12) in the vastus lateralis of the quadriceps and pectoralis muscle in horses being trained. In that study, three different insulin dependent GLUTs, more specifically GLUT4, GLUT8 and GLUT12 were mapped out for their evolution of expression in answer to 8 weeks of aerobic training and acute exercise. Basal GLUT4 and GLUT12 protein expression were significantly higher in locomotion *versus* posture muscles. Training had no effect on basal GLUT4 expression, neither in the VL, nor the PM. However, acute exercise in trained condition significantly decreased GLUT4 expression in the VL. Neither training nor acute exercise significantly changed total GLUT8 protein expression. Training significantly decreased total GLUT12 protein expression in rest biopsies, only visible in the VL. This decrease was even more prominent in the VL after acute exercise in trained condition (Vidal Moreno de Vega et al., 2023). These results question the importance of glucose as basal substrate to fuel training and exercise in healthy horses and are in line with previous research reporting persistently low glycogen levels and longer recovery time for muscle glycogen replenishment in trained horses compared to other mammals (Snow et al., 1983; Essén-Gustavsson et al., 1989; Lacombe et al., 2003; 2004).

In addition to its potential performance-enhancing effects, it would be valuable to explore the impact of aleurone supplementation in obese horses with equine metabolic syndrome. Research has shown that exercise alone is insufficient to reduce insulin resistance in these horses, and strict dietary restrictions are also necessary (Carter et al., 2010; Pratt-Phillips, 2024). Investigating the potential benefits and effects of incorporating aleurone into the diet of EMS-affected horses would be an interesting avenue for further study.

### Shifts in the fecal microbiome composition and predicted fecal metabolic output

The current study also looked at possible shifts that occurred in the gut microbiome of the horses involved, on one hand as a result of training, and on the other hand in response to aleurone supplementation. Using the PICRUSt software, changes in the metabolic output of the gut microbiome involved were also predicted in each case. The additional focus on the gut microbiome was mainly motivated by the results obtained in the previously published aleurone dosing trial, where it was suggested that aleurone may mediate its effects via a combined effect of factors such as 1) the effects of aleurone and its components on feed texture and subsequent digestive processing 2) the effects of aleurone and/or its components on metabolism and 3) microbiome composition and metabolic output.

The gut microbiome can be modulated by many factors such as probiotics, prebiotics, postbiotics, antibiotics, fecal microbiota transplantation (FMT), diet and exercise (Costa and Weese, 2012; Daly et al., 2012; Costa et al., 2015; Almeida et al., 2016; Schoster et al., 2016; Quigley and Gajula, 2020; Fava et al., 2022). Indeed, it is well known that exercise influences the gut microbiome in humans, mice, rats and dogs (Clarke et al., 2014; Evans et al., 2014; Kang et al., 2014; Lambert et al., 2015; Cook et al., 2016; Estaki et al., 2016; Petersen et al., 2017; Codella et al., 2018; Mohr et al., 2020; Zannoni et al., 2020; Bonomini-Gnutzmann et al., 2022; Boytar et al., 2023). Studies on the effects of exercise and training on the equine gut microbiome are scarce (Almeida et al., 2016; Janabi et al., 2016; 2017; Szemplinski et al., 2020; Górniak et al., 2021). But, it is clear that the gut microbiome plays an important role in digestion, energy metabolism, immunology and it is increasingly recognized as being crucial for maintaining good health in many species, including the horse (Gerber et al., 2012). Not only the composition but also the metabolic output of the gut microbiome can importantly influence the animal’s physiology and health status (Franzosa et al., 2019). Studies suggest a clear connection between the microbiota and the metabolic phenotype. For instance, when transplanting the gut microbiome of obese mice into lean germfree mice, these lean mice developed increased body mass and obesity-associated metabolic phenotypes such as broad scale skeletal muscle lipid accumulation. Cohoused twins of these germfree mice that were transplanted with the gut microbiome of lean co-twins on their turn, developed a lean body and healthy metabolic phenotype (Ridaura et al., 2013). Likewise, transplanting the gut microbiome of healthy mice into type 2 diabetes mellitus mice decreased insulin resistance and repaired pancreatic islet beta-cell function (Wang et al., 2020). However, these fecal transplant studies have yet to be confirmed in human subjects, replicated in rodents, or investigated in horses (Dalby, 2023). While obese horses exhibit a different fecal microbiome compared to lean individuals, whether this difference is a cause or an effect remains an intriguing area of research across all species (Biddle et al., 2018).

In the current study, both alpha diversity (community richness and phylogenetic diversity) and beta diversity (community dissimilarity) showed no significant changes in all tested conditions. It is entirely possible in a fecal microbiome study to observe no differences in alpha and beta diversity between groups while still detecting significant differences in bacterial populations at the genus level. Training without aleurone resulted in decreased abundance of *Pseudomonas* (genus level) and Pseudomonadaceae (family level) and decreased abundance of *Streptococcus* (genus level). The decrease in *Pseudomonas* could indicate a shift towards a more beneficial fecal microbial composition, given that *Pseudomonas* is often associated with infections and dysbiosis (Valentini et al., 2018). Aleurone supplementation combined with training showed a significant decrease in *Desulfovibrio* and Desulfovibrionaceae (genus and family level), which is notable as *Desulfovibrio* is often associated with inflammation in other species (Lindenberg et al., 2019; Chen et al., 2020). In horses trained without aleurone, decreased AIRg (acute insulin response to glucose) was observed, with a positive correlation to the abundance of Peptostreptococcaceae. This suggests that certain microbial changes might improve insulin sensitivity post-training. The correlation was stronger when aleurone was added, suggesting that the Peptostreptococcaceae family might play a role in modulating insulin sensitivity, particularly when aleurone is present. Therefore, training, both with and without aleurone, modulates the gut microbiome, but the effects of aleurone supplementation appear to be more pronounced in changing microbial diversity and metabolic pathways. Microbial changes, especially reductions in *Desulfovibrio* and correlations with Peptostreptococcaceae, may be linked to improved metabolic outcomes, particularly in terms of insulin sensitivity. Further research could explore how these microbial changes directly influence equine health, metabolism, and performance.

### Correlations between the fecal microbiome and metabolic health

The study also revealed significant correlations between specific bacterial genera and insulin and glucose metabolism parameters. For instance, horses trained without aleurone had a decrease in AIRg (acute insulin response to glucose) and were strongly positively correlated with Peptostreptococcaceae and *Marvinbryantia*, while a negative correlation was observed with *Paraprevotellaceae*. This correlation was even stronger when aleurone was added. These findings align with previous research indicating that certain bacterial genera can influence metabolic health. A higher abundance of Peptostreptococcaceae and *Marvinbryantia* are potentially linked to adverse metabolic outcomes (Naderpoor et al., 2019). Additionally, IS changes correlated positively with the abundance of Ruminococcaceae and *Victivallis*, and negatively with Peptostreptococcaceae and *Marvinbryantia*. This suggests a complex interplay between gut microbiota and host metabolic health, where specific bacterial taxa may either exacerbate or ameliorate insulin resistance and glucose intolerance.

Studies on the impact of exercise and training on the equine gut microbiome are limited (Almeida et al., 2016; Janabi et al., 2016; 2017; Szemplinski et al., 2020). Janabi et al. (2017) found no effect of acute anaerobic exercise on microbial diversity or relative abundance but reported transient increases in Spirochaetes, Proteobacteria (now Pseudomonadota), and Bacteroidetes after 12 weeks of training. Bacterial diversity initially declined but returned to baseline by week six (Janabi et al., 2016). Szemplinski et al. (2020) similarly observed no changes in fecal microbiome 48 h post-exercise. Almeida et al. (2016) reported significant shifts in fecal beta diversity after 42 days of aerobic training but no alterations in alpha diversity. In conditioned fillies, acute exercise reduced the relative abundance of Chlamydiae and *Mycobacterium*, whereas untrained horses showed no significant microbiome changes.

Recently, the difference in fecal microbiome between insulin sensitive and insulin resistant ponies was described (Fitzgerald et al., 2020). Medium-insulin dysregulated (MID) ponies had higher fecal phylum Firmicutes levels than severe-insulin dysregulated (SID) ponies and normal insulin regulated ponies (NID). Both MID and SIG groups exhibited decreased abundance of taxa *Ruminococcacaea,* Lachnospiraceae*,* Rikenellaceae*,* Christensenellaceae and *Saccharimonadaceae* compared to normal ponies. Alpha diversity parameter evenness was significantly lower for MID ponies compared to NID and SID ponies. SID ponies showed lower alpha diversity than NID but the difference was not significant. Insulin dysregulation did not influence beta diversity.

Keeping the aforementioned in mind: Aleurone supplementation led to shifts in fecal microbiome composition and predicted metabolic pathways, beyond the effects of training alone. While training reduced the abundance of potentially dysbiotic genera such as *Pseudomonas* and *Streptococcus*, aleurone supplementation was associated with decreases in *Desulfovibrio*, a genus linked to inflammation. Positive correlations between reduced Peptostreptococcaceae and enhanced insulin sensitivity further emphasize the role of the gut microbiome in mediating metabolic improvements.

The lack of significant changes in microbial diversity metrics underscores that functional shifts in the microbiome, rather than taxonomic richness, may drive the observed metabolic benefits. Predictive metagenomic analyses suggest that aleurone-induced changes in the microbiome may improve energy metabolism and help reduce inflammatory processes, which aligns with its observed effects on insulin dynamics.

Our research group recently reported on xenobiotics found in muscle biopsies taken from Friesian horses trained on a treadmill. It was hypothesized that these xenobiotics are derived from the horse gut’s microbiome and are used in the muscle energy metabolism, the latter underscores the importance of the gut microbiome composition and metabolic output on overall equine metabolism (De Meeûs D’Argenteuil et al., 2021).

## Conclusion

This study showed aleurone supplementation as a promising dietary strategy to complement training in horses, providing measurable benefits for metabolic health and performance capacity. These findings are especially relevant for equine athletes, where optimized glucose metabolism is important during strategic accelerating phases in competition. Additionally, exploring aleurone’s application in metabolically challenged horses, such as those suffering from Equine Metabolic Syndrome, could further elucidate its therapeutic potential.

Future research should investigate aleurone’s mechanistic role at the level of skeletal muscle and its broader implications for systemic metabolism. Additionally, long-term studies assessing performance outcomes in competitive settings will help validate the potential practical benefits of aleurone supplementation in athletic horses. These findings highlight the potential of dietary interventions in improving metabolic health and performance in horses, most probably suitable to be extrapolated to other species including humans.

## Data Availability

TThe datasets presented in this study can be found in online repositories. The names of the repository/repositories and accession number(s) can be found below: https://figshare.com/, 10.6084/m9.figshare.28254374

## References

[B1] Almeida R. Gerbaba T. Petrof E. O. (2016). Recurrent Clostridium difficile infection and the microbiome. J. Gastroenterol. 51, 1–10. 10.1007/s00535-015-1099-3 26153514

[B2] Arfuso F. Assenza A. Fazio F. Rizzo M. Giannetto C. Piccione G. (2019). Dynamic change of serum levels of some branched-chain amino acids and tryptophan in athletic horses after different physical exercises. J. Equine Vet. Sci. 77, 12–16. 10.1016/j.jevs.2019.02.006 31133304

[B3] Asplin K. E. Sillence M. N. Pollitt C. C. McGowan C. M. (2007). Induction of laminitis by prolonged hyperinsulinaemia in clinically normal ponies. Veterinary J. 174, 530–535. 10.1016/j.tvjl.2007.07.003 17719811

[B4] Assenza A. Arfuso F. Fazio F. Giannetto C. Rizzo M. Zumbo A. (2018). Effect of gender and jumping exercise on leukocyte number, dopamine and prolactin levels in horses. Thai J. Veterinary Med. 48, 95–101. 10.56808/2985-1130.2895

[B5] Biddle A. S. Tomb J. F. Fan Z. (2018). Microbiome and blood analyte differences point to community and metabolic signatures in lean and obese horses. Front. Vet. Sci. 5, 225. 10.3389/fvets.2018.00225 30294603 PMC6158370

[B6] Bohm A. Bogoni C. Behrens R. Otto T. (2003). Method for the extraction of aleurone from bran. Available online at: https://patents.google.com/patent/US20030175384A1/en (Accessed March 21, 2025).

[B7] Bolyen E. Rideout J. R. Dillon M. R. Bokulich N. A. Abnet C. C. Al-Ghalith G. A. (2019). Reproducible, interactive, scalable and extensible microbiome data science using QIIME 2. Nat. Biotechnol. 37, 852–857. 10.1038/s41587-019-0209-9 31341288 PMC7015180

[B8] Bonomini-Gnutzmann R. Plaza-Díaz J. Jorquera-Aguilera C. Rodríguez-Rodríguez A. Rodríguez-Rodríguez F. (2022). Effect of intensity and duration of exercise on gut microbiota in humans: a systematic review. Res. Public Health 19, 9518. 10.3390/ijerph PMC9368618 35954878

[B9] Borghouts L. B. Backx K. Mensink M. F. Keizer H. A. (1999). Effect of training intensity on insulin sensitivity as evaluated by insulin tolerance test. Eur. J. Appl. Physiol. Occup. Physiol. 80, 461–466. 10.1007/s004210050618 10502080

[B10] Boshuizen B. Moreno de Vega C. V. De Maré L. de Meeûs C. de Oliveira J. E. Hosotani G. (2021). Effects of aleurone supplementation on glucose-insulin metabolism and gut microbiome in untrained healthy horses. Front. Vet. Sci. 8, 642809. 10.3389/fvets.2021.642809 33912605 PMC8072273

[B11] Boston R. C. Stefanovski D. Moate P. J. Sumner A. E. Watanabe R. M. Bergman R. N. (2003). MINMOD Millennium: a computer program to calculate glucose effectiveness and insulin sensitivity from the frequently sampled intravenous glucose tolerance test. Diabetes Technol. Ther. 5, 1003–1015. 10.1089/152091503322641060 Available online at: www.liebertpub.com. 14709204

[B12] Boytar A. N. Skinner T. L. Wallen R. E. Jenkins D. G. Dekker Nitert M. (2023). The effect of exercise prescription on the human gut microbiota and comparison between clinical and apparently healthy populations: a systematic review. Nutrients 15, 1534. 10.3390/nu15061534 36986264 PMC10054511

[B13] Brouns F. Youna H. Ruth P. Anson N. M. (2012). Wheat aleurone: separation, composition, health aspects, and potential food use. Crit. Rev. Food Sci. Nutr. 52, 553–568. 10.1080/10408398.2011.589540 22452734

[B14] Caporaso J. G. Kuczynski J. Stombaugh J. Bittinger K. Bushman F. D. Costello E. K. (2010). QIIME allows analysis of high-throughput community sequencing data. Nat. Methods 7, 335–336. 10.1038/nmeth.f.303 20383131 PMC3156573

[B15] Carter R. A. McCutcheon L. J. Valle E. Meilahn E. N. Geor R. J. (2010). Effects of exercise training on adiposity, insulin sensitivity, and plasma hormone and lipid concentrations in overweight or obese, insulin-resistant horses. Am. J. Vet. Res. 71, 314–321. 10.2460/ajvr.71.3.314 20187833

[B16] Caspi R. Billington R. Keseler I. M. Kothari A. Krummenacker M. Midford P. E. (2020). The MetaCyc database of metabolic pathways and enzymes-a 2019 update. Nucleic Acids Res. 48, D445–D453. 10.1093/nar/gkz862 31586394 PMC6943030

[B17] Chen L. Collij V. Jaeger M. van den Munckhof I. C. L. Vich Vila A. Kurilshikov A. (2020). Gut microbial co-abundance networks show specificity in inflammatory bowel disease and obesity. Nat. Commun. 11, 4018. 10.1038/s41467-020-17840-y 32782301 PMC7419557

[B18] Clarke S. F. Murphy E. F. O’Sullivan O. Lucey A. J. Humphreys M. Hogan A. (2014). Exercise and associated dietary extremes impact on gut microbial diversity. Gut 63, 1913–1920. 10.1136/gutjnl-2013-306541 25021423

[B19] Codella R. Luzi L. Terruzzi I. (2018). Exercise has the guts: how physical activity may positively modulate gut microbiota in chronic and immune-based diseases. Dig. Liver Dis. 50, 331–341. 10.1016/j.dld.2017.11.016 29233686

[B20] Cook M. D. Allen J. M. Pence B. D. Wallig M. A. Gaskins H. R. White B. A. (2016). Exercise and gut immune function: evidence of alterations in colon immune cell homeostasis and microbiome characteristics with exercise training. Immunol. Cell Biol. 94, 158–163. 10.1038/icb.2015.108 26626721

[B21] Costa M. C. Stämpfli H. R. Arroyo L. G. Allen-Vercoe E. Gomes R. G. Weese J. S. (2015). Changes in the equine fecal microbiota associated with the use of systemic antimicrobial drugs. BMC Vet. Res. 11, 19. 10.1186/s12917-015-0335-7 25644524 PMC4323147

[B22] Costa M. C. Weese J. S. (2012). The equine intestinal microbiome. Anim. Health Res. Rev. 13, 121–128. 10.1017/S1466252312000035 22626511

[B23] Costabile G. Vitale M. Della Pepa G. Cipriano P. Vetrani C. Testa R. (2022). A wheat aleurone-rich diet improves oxidative stress but does not influence glucose metabolism in overweight/obese individuals: results from a randomized controlled trial. Nutr. Metabolism Cardiovasc. Dis. 32, 715–726. 10.1016/j.numecd.2021.12.016 35123855

[B24] Dalby M. J. (2023). Questioning the foundations of the gut microbiota and obesity. Philosophical Trans. R. Soc. B Biol. Sci. 378, 20220221. 10.1098/rstb.2022.0221 PMC10475866 37661739

[B25] Daly K. Proudman C. J. Duncan S. H. Flint H. J. Dyer J. Shirazi-Beechey S. P. (2012). Alterations in microbiota and fermentation products in equine large intestine in response to dietary variation and intestinal disease. Br. J. Nutr. 107, 989–995. 10.1017/S0007114511003825 21816118

[B26] de Laat M. A. McGree J. M. Sillence M. N. (2015). Equine hyperinsulinemia: Investigation of the enteroinsular axis during insulin dysregulation. Am. J. Physiol. Endocrinol. Metab. 310, E61–E72. 10.1152/ajpendo.00362.2015 26530154

[B27] De Maré L. Boshuizen B. Vidal Moreno de Vega C. de Meeûs C. Plancke L. Gansemans Y. (2022). Profiling the aerobic window of horses in response to training by means of a modified lactate minimum speed test: flatten the curve. Front. Physiol. 13, 792052. 10.3389/fphys.2022.792052 35392373 PMC8982777

[B28] De Meeûs D’Argenteuil C. Boshuizen B. Oosterlinck M. Van De Winkel D. De Spiegelaere W. De Bruijn C. M. (2021). Flexibility of equine bioenergetics and muscle plasticity in response to different types of training: an integrative approach, questioning existing paradigms. PLoS One 16, e0249922. 10.1371/journal.pone.0249922 33848308 PMC8043414

[B29] DeSantis T. Z. Hugenholtz P. Larsen N. Rojas M. Brodie E. L. Keller K. (2006). Greengenes, a chimera-checked 16S rRNA gene database and workbench compatible with ARB. Appl. Environ. Microbiol. 72, 5069–5072. 10.1128/AEM.03006-05 16820507 PMC1489311

[B30] Duncan G. E. Perri M. G. Theriaque D. W. Hutson A. D. Eckel R. H. Stacpoole P. W. (2003). Exercise training, without weight loss, increases insulin sensitivity and postheparin plasma lipase activity in previously sedentary adults. Diabetes Care 26, 557–562. 10.2337/diacare.26.3.557 12610001

[B31] Durham A. E. Frank N. McGowan C. M. Menzies-Gow N. J. Roelfsema E. Vervuert I. (2019). ECEIM consensus statement on equine metabolic syndrome. J. Vet. Intern Med. 33, 335–349. 10.1111/jvim.15423 30724412 PMC6430910

[B32] Essén‐Gustavsson B. McMiken D. Karlström K. Lindholm A. Persson S. Thornton J. (1989). Muscular adaptation of horses during intensive training and detraining. Equine Vet. J. 21, 27–33. 10.1111/j.2042-3306.1989.tb02085.x 2920697

[B33] Estaki M. Pither J. Baumeister P. Little J. P. Gill S. K. Ghosh S. (2016). Cardiorespiratory fitness as a predictor of intestinal microbial diversity and distinct metagenomic functions. Microbiome 4, 42. 10.1186/s40168-016-0189-7 27502158 PMC4976518

[B34] Evans C. C. LePard K. J. Kwak J. W. Stancukas M. C. Laskowski S. Dougherty J. (2014). Exercise prevents weight gain and alters the gut microbiota in a mouse model of high fat diet-induced obesity. PLoS One 9, e92193. 10.1371/journal.pone.0092193 24670791 PMC3966766

[B35] Fava F. Ulaszewska M. M. Scholz M. Stanstrup J. Nissen L. Mattivi F. (2022). Impact of wheat aleurone on biomarkers of cardiovascular disease, gut microbiota and metabolites in adults with high body mass index: a double-blind, placebo-controlled, randomized clinical trial. Eur. J. Nutr. 61, 2651–2671. 10.1007/s00394-022-02836-9 35247098 PMC9279244

[B90] Filho H. C. M. Manso H. E. C. C. C. Watford M. McKeever K. H. (2020). Abundance of the skeletal muscle Glut-4 glucose transport protein in Standardbred foals during development and exercise. Comp. Exerc. Physiol. 16, 395–402. 10.3920/CEP200008

[B37] Fitzgerald D. M. Spence R. J. Stewart Z. K. Prentis P. J. Sillence M. N. De Laat M. A. (2020). The effect of diet change and insulin dysregulation on the faecal microbiome of ponies. J. Exp. Biol. 223, jeb219154. 10.1242/jeb.219154 32098884

[B38] Frank N. Geor R. (2014). Current best practice in clinical management of equine endocrine patients. Equine Vet. Educ. 26, 6–9. 10.1111/eve.12130

[B39] Franzosa E. A. Sirota-Madi A. Avila-Pacheco J. Fornelos N. Haiser H. J. Reinker S. (2019). Gut microbiome structure and metabolic activity in inflammatory bowel disease. Nat. Microbiol. 4, 293–305. 10.1038/s41564-018-0306-4 30531976 PMC6342642

[B40] García-Estévez D. A. Araújo-Vilar D. Fiestras-Janeiro G. Saavedra-González Á. Cabezas-Cerrato J. (2003). Comparison of several insulin sensitivity indices derived from basal plasma insulin and glucose levels with minimal model indices. Hormone Metabolic Res. 35, 13–17. 10.1055/s-2003-38385 12669265

[B41] Gerber G. K. Onderdonk A. B. Bry L. (2012). Inferring dynamic signatures of microbes in complex host ecosystems. PLoS Comput. Biol. 8, e1002624. 10.1371/journal.pcbi.1002624 22876171 PMC3410865

[B42] Górniak W. Cholewińska P. Szeligowska N. Wołoszyńska M. Soroko M. Czyż K. (2021). Effect of intense exercise on the level of bacteroidetes and firmicutes phyla in the digestive system of thoroughbred racehorses. Animals 11, 290–299. 10.3390/ANI11020290 33498857 PMC7910997

[B44] Henneke D. R. Potter G. D. Kreider J. L. Yeates B. F. (1983). Relationship between condition score, physical measurements and body fat percentage in mares. Equine Vet. J. 15, 371–372. 10.1111/j.2042-3306.1983.tb01826.x 6641685

[B91] Ihaka R. Gentleman R. (1996). R: A language for data analysis and graphics. J. Comput. Graph. Stat. 5, 299–314. 10.1080/10618600.1996.10474713

[B45] Janabi A. Biddle A. Klein D. McKeever K. (2016). Exercise training-induced changes in the gut microbiota of Standardbred racehorses. Comp. Exerc Physiol. 12, 119–130. 10.3920/CEP160015

[B46] Janabi A. H. D. Biddle A. S. Klein D. J. McKeever K. H. (2017). The effects of acute strenuous exercise on the faecal microbiota in Standardbred racehorses. Comp. Exerc Physiol. 13, 13–24. 10.3920/CEP160030

[B47] Jeffcott L. B. Field J. R. McLean J. G. O’Dea K. (1986). Glucose tolerance and insulin sensitivity in ponies and Standardbred horses. Equine Vet. J. 18, 97–101. 10.1111/j.2042-3306.1986.tb03556.x 3516677

[B48] Kang S. S. Jeraldo P. R. Kurti A. Miller M. E. B. Cook M. D. Whitlock K. (2014). Diet and exercise orthogonally alter the gut microbiome and reveal independent associations with anxiety and cognition. Mol. Neurodegener. 9, 36. 10.1186/1750-1326-9-36 25217888 PMC4168696

[B49] Karikoski N. P. McGowan C. M. Singer E. R. Asplin K. E. Tulamo R. M. Patterson-Kane J. C. (2015). Pathology of natural cases of equine endocrinopathic laminitis associated with hyperinsulinemia. Vet. Pathol. 52, 945–956. 10.1177/0300985814549212 25232034

[B50] Keaveney E. M. Price R. K. Hamill L. L. Wallace J. M. W. McNulty H. Ward M. (2015). Postprandial plasma betaine and other methyl donor-related responses after consumption of minimally processed wheat bran or wheat aleurone, or wheat aleurone incorporated into bread. Br. J. Nutr. 113, 445–453. 10.1017/S0007114514003778 25585164

[B51] Lacombe V. A. Hinchcliff K. W. Kohn C. W. Devor S. T. Taylor L. E. (2004). Effects of feeding meals with various soluble-carbohydrate content on muscle glycogen synthesis after exercise in horses. Am. J. Vet. Res. 65, 916–923. 10.2460/ajvr.2004.65.916 15281649

[B52] Lacombe V. A. Hinchcliff K. W. Taylor L. E. (2003). Interactions of substrate availability, exercise performance, and nutrition with muscle glycogen metabolism in horses. J. Am. Vet. Med. Assoc. 223, 1576–1585. 10.2460/javma.2003.223.1576 14664443

[B53] Lambert J. E. Myslicki J. P. Bomhof M. R. Belke D. D. Shearer J. Reimer R. A. (2015). Exercise training modifies gut microbiota in normal and diabetic mice. Appl. Physiology, Nutr. Metabolism 40, 749–752. 10.1139/apnm-2014-0452 25962839

[B54] Langille M. G. I. Zaneveld J. Caporaso J. G. McDonald D. Knights D. Reyes J. A. (2013). Predictive functional profiling of microbial communities using 16S rRNA marker gene sequences. Nat. Biotechnol. 31, 814–821. 10.1038/nbt.2676 23975157 PMC3819121

[B56] Leschke D. H. Muir G. S. Hodgson J. K. Coyle M. Horn R. Bertin F. R. (2019). Immunoreactive insulin stability in horses at risk of insulin dysregulation. J. Vet. Intern Med. 33, 2746–2751. 10.1111/jvim.15629 31617618 PMC6872612

[B57] Lindenberg F. Krych L. Kot W. Fielden J. Frøkiær H. van Galen G. (2019). Development of the equine gut microbiota. Sci. Rep. 9, 14427. 10.1038/s41598-019-50563-9 31594971 PMC6783416

[B58] Mandal S. Van Treuren W. White R. A. Eggesbø M. Knight R. Peddada S. D. (2015). Analysis of composition of microbiomes: a novel method for studying microbial composition. Microb. Ecol. Health Dis. 26, 27663. 10.3402/mehd.v26.27663 26028277 PMC4450248

[B59] Manso Filho H. C. Betros C. L. Gordon M. E. Manso H. E. C. C. C. Watford M. McKeever K. H. (2017). Exercise training, Glut-4 protein abundance and glutamine in skeletal muscle of mature and very old horses. Comp. Exerc Physiol. 13, 63–69. 10.3920/CEP170003

[B60] McCutcheon L. J. Geor R. J. Hinchcliff K. W. (2002). Changes in skeletal muscle GLUT4 content and muscle membrane glucose transport following 6 weeks of exercise training. Equine Vet. J. Suppl. 34, 199–204. 10.1111/j.2042-3306.2002.tb05418.x 12405686

[B61] Menzies-Gow N. J. Wray H. Bailey S. R. Harris P. A. Elliott J. (2014). The effect of exercise on plasma concentrations of inflammatory markers in normal and previously laminitic ponies. Equine Vet. J. 46, 317–321. 10.1111/evj.12132 23819851

[B62] Mohr A. E. Jäger R. Carpenter K. C. Kerksick C. M. Purpura M. Townsend J. R. (2020). The athletic gut microbiota. J. Int. Soc. Sports Nutr. 17, 24. 10.1186/s12970-020-00353-w 32398103 PMC7218537

[B63] Naderpoor N. Mousa A. Gomez-Arango L. F. Barrett H. L. Nitert M. D. Courten B.De (2019). Faecal microbiota are related to insulin sensitivity and secretion in overweight or obese adults. J. Clin. Med. 8, 452. 10.3390/jcm8040452 30987356 PMC6518043

[B64] Neyrinck A. M. De Backer F. Cani P. D. Bindels L. B. Stroobants A. Portetelle D. (2008). Immunomodulatory properties of two wheat bran fractions - aleurone-enriched and crude fractions - in obese mice fed a high fat diet. Int. Immunopharmacol. 8, 1423–1432. 10.1016/j.intimp.2008.05.015 18687304

[B65] Nout Y. S. Hinchcliff K. W. Jose-Cunilleras E. Dearth L. R. Sivko G. S. DeWille J. W. (2003). Effect of moderate exercise immediately followed by induced hyperglycemia on gene expression and content of the glucose transporter-4 protein in skeletal muscles of horses. Am. J. Vet. Res. 64, 1401–1408. 10.2460/ajvr.2003.64.1401 14620777

[B66] Pacini G. Bergman R. N. (1986). MINMOD: a computer program to calculate insulin sensitivity and pancreatic responsivity from the frequently sampled intravenous glucose tolerance test. Comput. Methods Programs Biomed. 23, 113–122. 10.1016/0169-2607(86)90106-9 3640682

[B67] Parks D. H. Tyson G. W. Hugenholtz P. Beiko R. G. (2014). STAMP: statistical analysis of taxonomic and functional profiles. Bioinformatics 30, 3123–3124. 10.1093/bioinformatics/btu494 25061070 PMC4609014

[B69] Petersen L. M. Bautista E. J. Nguyen H. Hanson B. M. Chen L. Lek S. H. (2017). Community characteristics of the gut microbiomes of competitive cyclists. Microbiome 5, 98. 10.1186/s40168-017-0320-4 28797298 PMC5553673

[B70] Piccione G. Giannetto C. Faggio C. Alberghina D. Panzera M. (2013). Three-time feeding does not influence insulin daily rhythm in horses. Biol. Rhythm Res. 44, 421–426. 10.1080/09291016.2012.692258

[B71] Pratt S. E. Geor R. J. Spriet L. L. McCutcheon L. J. (2007). Time course of insulin sensitivity and skeletal muscle glycogen synthase activity after a single bout of exercise in horses. J. Appl. Physiol. 103, 1063–1069. 10.1152/japplphysiol.01349.2006 17585040

[B72] Pratt-Phillips S. (2024). Effect of exercise conditioning on countering the effects of obesity and insulin resistance in horses—a review. Animals 14, 727. 10.3390/ani14050727 38473112 PMC10931081

[B73] Pratt-Phillips S. E. Geor R. J. McCutcheon L. J. (2015). Comparison among the euglycemic-hyperinsulinemic clamp, insulin-modified frequently sampled intravenous glucose tolerance test, and oral glucose tolerance test for assessment of insulin sensitivity in healthy Standardbreds. Am. J. Vet. Res. 76, 84–91. 10.2460/ajvr.76.1.84 25535665

[B74] Pritchard A. Nielsen B. McLean A. Robison C. Yokoyama M. Hengemuehle S. (2019). Insulin resistance as a result of body condition categorized as thin, moderate, and obese in domesticated U.S. donkeys (*Equus asinus*). J. Equine Vet. Sci. 77, 31–35. 10.1016/j.jevs.2019.02.011 31133313

[B75] Quigley E. M. M. Gajula P. (2020). Recent advances in modulating the microbiome. Res 9, F1000. 10.12688/f1000research.20204.1 PMC6993818 32047611

[B92] Ridaura V. K. Faith J. J. Rey F. E. Cheng J. Duncan A. E. Kau A. L. (2013). Gut microbiota from twins discordant for obesity modulate metabolism in mice. Sci. 1979, 341. 10.1126/science.1241214 PMC3829625 24009397

[B76] Rosa N. N. Pekkinen J. Zavala K. Fouret G. Korkmaz A. Feillet-Coudray C. (2014). Impact of wheat aleurone structure on metabolic disorders caused by a high-fat diet in mice. J. Agric. Food Chem. 62, 10101–10109. 10.1021/jf503314a 25238637

[B77] Schoster A. Mosing M. Jalali M. Staempfli H. R. Weese J. S. (2016). Effects of transport, fasting and anaesthesia on the faecal microbiota of healthy adult horses. Equine Vet. J. 48, 595–602. 10.1111/evj.12479 26122549

[B78] Slentz C. A. Tanner C. J. Bateman L. A. Durheim M. T. Huffman K. M. Houmard J. A. (2009). Effects of exercise training intensity on pancreatic β-cell function. Diabetes Care 32, 1807–1811. 10.2337/dc09-0032 19592624 PMC2752909

[B79] Smith S. Harris P. A. Menzies-Gow N. J. (2016). Comparison of the in-feed glucose test and the oral sugar test. Equine Vet. J. 48, 224–227. 10.1111/evj.12413 25582152

[B80] Snow D. H. Persson S. G. B. Rose R. J. (1983). “Equine exercise physiology,” in Proceedings of the first international conference, Oxford, September 22nd-24th, 1982. Cambridge. Granta Editions, 47 Norfolk Street.

[B81] Stewart-Hunt L. Geor R. J. Mccutcheon L. J. (2006). Effects of short-term training on insulin sensitivity and skeletal muscle glucose metabolism in Standardbred horses. Equine Vet. J. 38, 226–232. 10.1111/j.2042-3306.2006.tb05544.x 17402423

[B82] Szemplinski K. L. Thompson A. Cherry N. Guay K. Smith W. B. Brady J. (2020). Transporting and exercising unconditioned Horses: effects on microflora populations. J. Equine Vet. Sci. 90, 102988. 10.1016/j.jevs.2020.102988 32534767

[B83] Valberg S. J. Velez-Irizarry D. Williams Z. J. Pagan J. D. Mesquita V. Waldridge B. (2023). Novel expression of GLUT3, GLUT6 and GLUT10 in equine gluteal muscle following glycogen-depleting exercise: impact of dietary starch and fat. Metabolites 13, 718. 10.3390/metabo13060718 37367876 PMC10301051

[B84] Valentini M. Gonzalez D. Mavridou D. A. Filloux A. (2018). Lifestyle transitions and adaptive pathogenesis of *Pseudomonas aeruginosa* . Curr. Opin. Microbiol. 41, 15–20. 10.1016/j.mib.2017.11.006 29166621

[B85] Vidal Moreno de Vega C. Lemmens D. de Meeûs d’Argenteuil C. Boshuizen B. de Maré L. Leybaert L. (2023). Dynamics of training and acute exercise-induced shifts in muscular glucose transporter (GLUT) 4, 8, and 12 expression in locomotion versus posture muscles in healthy horses. Front. Physiol. 14, 1256217. 10.3389/fphys.2023.1256217 37654675 PMC10466803

[B86] Walsh D. M. McGowan C. M. McGowan T. Lamb S. V. Schanbacher B. J. Place N. J. (2009). Correlation of plasma insulin concentration with laminitis score in a field study of equine cushing’s disease and equine metabolic syndrome. J. Equine Vet. Sci. 29, 87–94. 10.1016/j.jevs.2008.12.006

[B87] Wang H. Lu Y. Yan Y. Tian S. Zheng D. Leng D. (2020). Promising treatment for type 2 diabetes: fecal microbiota transplantation reverses insulin resistance and impaired islets. Front. Cell Infect. Microbiol. 9, 455. 10.3389/fcimb.2019.00455 32010641 PMC6979041

[B88] Zannoni A. Pietra M. Gaspardo A. Accorsi P. A. Barone M. Turroni S. (2020). Non-invasive assessment of fecal stress biomarkers in hunting dogs during exercise and at rest. Front. Vet. Sci. 7, 126. 10.3389/fvets.2020.00126 32373631 PMC7186473

[B89] Zhang H. Chen X. Braithwaite D. He Z. (2014). Phylogenetic and metagenomic analyses of substrate-dependent bacterial temporal dynamics in microbial fuel cells. PLoS One 9, e107460. 10.1371/journal.pone.0107460 25202990 PMC4159341

