# Supplementary material for: Aleurone supplementation enhances the metabolic benefits of training in Standardbred mares: impacts on glucose-insulin dynamics and gut microbiome composition
Source: Front Physiol. 2025 Apr 10;16:1565005. doi: 10.3389/fphys.2025.1565005 (PMC12018385; doi:10.3389/fphys.2025.1565005)
Supplement: Supplementary file 1 [file DataSheet1.pdf]

Supplementary Material

1 Supplementary Figures and Tables

Supplementary Figures

1.1

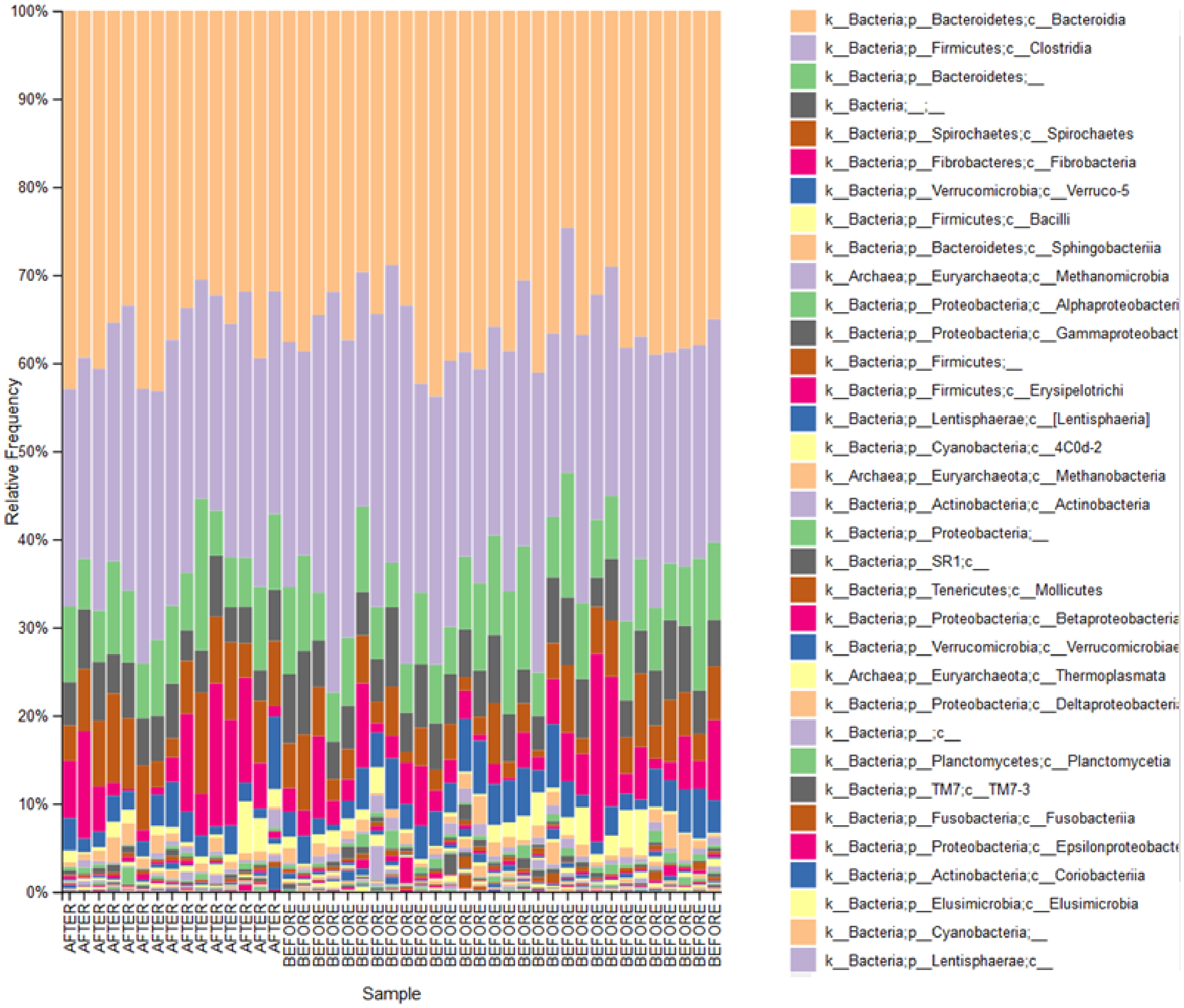

Supplementary Figure 1. Taxa bar plot: Percentages of total identifiable reads for prominent bacterial classes identified following sequencing of fecal bacterial DNA. Each bar represents one horse's sample either 'After' 8 weeks of aleurone supplementation and training or 'Before'. The key notes the dominant bacterial classes represented by each colored bar.

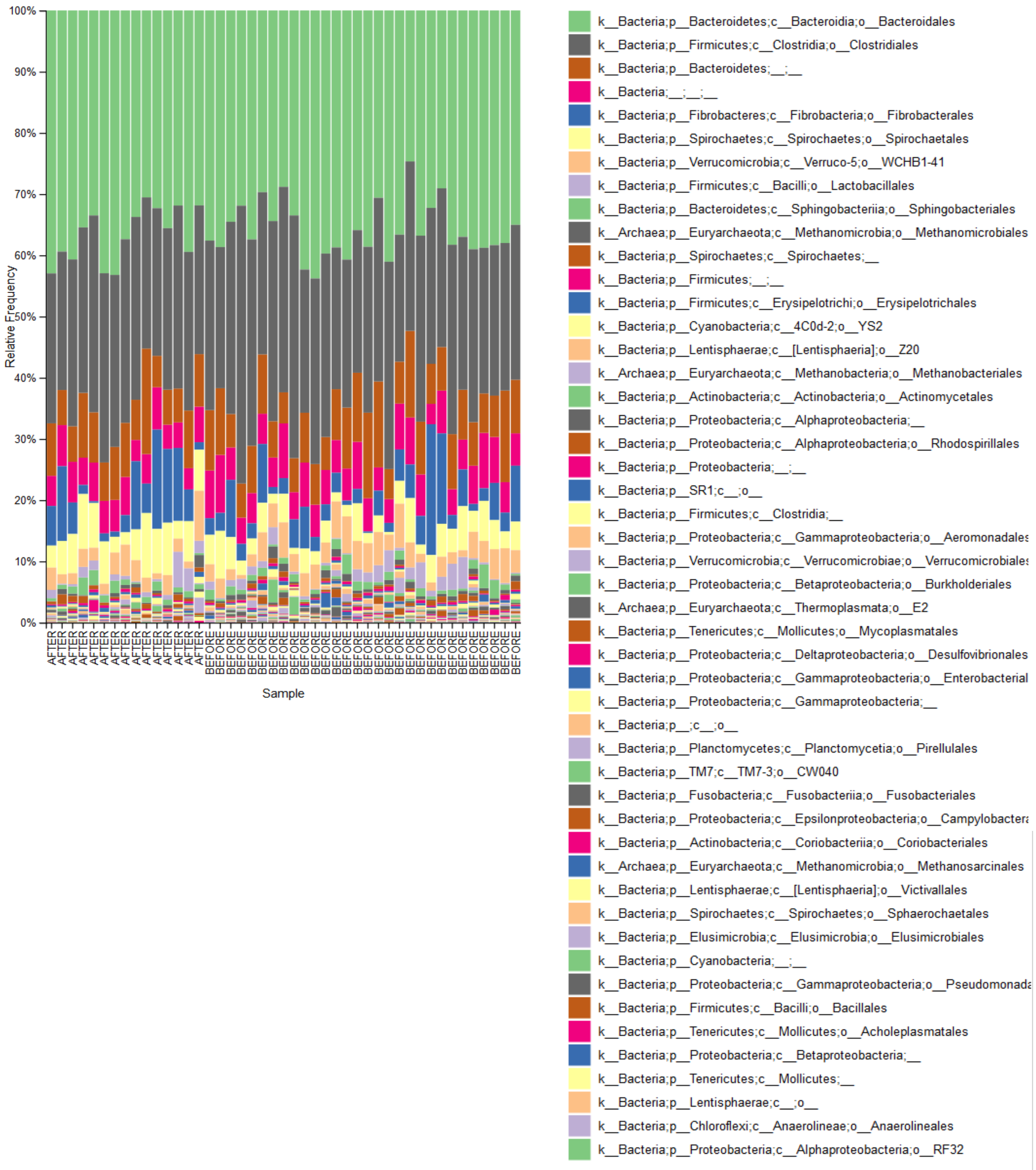

Supplementary Figure 2. Taxa bar plot: Percentages of total identifiable reads for prominent bacterial orders identified following sequencing of fecal bacterial DNA. Each bar represents one fecal sample either 'After' 8 weeks of aleurone supplementation and training or 'Before' training and aleurone supplementation. The key notes the dominant bacterial orders represented by each colored bar.

1.2 Supplementary Tables

Supplementary Table 1.

Comparative feed analysis of the macronutrient composition of both the Blanco feed and the aleurone concentrate used in the study.

Blanco feed	
**Raw materials**	**Amount (%)**
Wheat short flour	29,982
Barley	21,000
Soybeans 30% RC	11,000
Alfalfa for horses	10,500
Fine corn flakes RV 11	8,000
Wheat nt. Grain (flour)	6,525
Molasses P67	4,000
Palm oil	2,475
Fine chalk	2,275
Maize	2,000
Salt	0,900
Dry matter MD	0,500
Premix horse 0,25%	0,400
Sodium bicarbonate	0,288
MgO 90%	0,125
Vit E 50%	0,030
Total	100,000
**Nutrients**	**Amount in product**
Dry matter	875,982
Moisture	124,018
Crude ash	77724,000
Crude protein	111,260
Crude fat	55,068
Crude fiber	105,091
Other carbohydrates	524,069
Starch	282,780
Sugars	59,396

Sugars and starch	342,123
Horse Feed Unit	825,987
EWpa	0,701
FOS g/kg	497,487
Digestible crude protein	85,562
Lysine	4,638
Methionine	1,789
NSP g/kg	312,479
Na+ K-Cl meq/kg	270,116
Calcium	12,449
Potassium	9,798
Sodium	4,505
Chlorine	6,408
Magnesium	2,627
Total phosphorus	4,315
Selenium (mg/kg)	0,544
Sodium selenite (E8)/selenium	0,544
Iron (mg/kg)	340,291
Iron (II) sulfate monohydrate (E1)/iron	200,000
Manganese (mg/kg)	119,176
Manganese (II) oxide (E5)/manganese	80,000
Zinc (mg/kg)	158,944
Zinc oxide (E6)/zinc	120,000
Copper (mg/kg)	39,504
Copper (II) sulfate pentahydrate (E4)/copper	34,000
Iodine (mg/kg)	1,059
Anhydrous calcium iodate (E2)/iodine	1,000
Cobalt (mg/kg)	0,891
Cobalt (II) carbinate hydroxide (2:3) monohydrate	0,000
C18:2 g/kg	14,567
C18:3 g/kg	1,327
Vitamin A (E672)	25000,000
Vitamin D3 (E671)	3000,000

Vitamin E (All-rac-alpha-tocopheryl acetate)	150,000
Vitamin K3	4,000
Vitamin B1	20,000
Vitamin B2	25,000
Calcium-D-Pantothenate	30,000
Vitamin B6	10,000
Vitamin B9	4,000
Vitamin B12	64,000
Vitamin PP (Niacin)	60,000
Vitamin C	0,000
Biotin	304,000

**Aleurone concentrate**	
**Raw materials**	**Amount (%)**
Wheat aleurone	20,000
Barley	20,045
Soybeans 30% RC	12,375
Alfalfa for horses	10,500
Fine corn flakes RV 11	8,000
Wheat short flour	7,100
Wheat nt. Grain (flour)	4,775
Molasses P67	4,000
Palm oil	2,425
Fine chalk	2,300
Maize	6,325
Salt	0,900
Dry matter MD	0,500
Premix horse 0,25%	0,400
Sodium bicarbonate	0,200
MgO 90%	0,125
Vit E 50%	0,030
Total	100,000
**Nutrients**	**Amount in product**

Dry matter	876,333
Moisture	123,667
Crude ash	76,651
Crude protein	113,603
Crude fat	54,997
Crude fiber	110,350
Other carbohydrates	525,339
Starch	260,134
Sugars	55,735
Sugars and starch	315,834
Horse Feed Unit	830,920
EWpa	0,708
FOS g/kg	493,734
Digestible crude protein	83,382
Lysine	4,567
Methionine	1,770
NSP g/kg	312,386
Na+ K-Cl meq/kg	255,069
Calcium	12,499
Potassium	10,423
Sodium	4,581
Chlorine	6,425
Magnesium	3,325
Total phosphorus	5,312
Selenium (mg/kg)	0,544
Sodium selenite (E8)/selenium	0,544
Iron (mg/kg)	340,559
Iron (II) sulfate monohydrate (E1)/iron	200,000
Manganese (mg/kg)	116,138
Manganese (II) oxide (E5)/manganese	80,000
Zinc (mg/kg)	157,778
Zinc oxide (E6)/zinc	120,000
Copper (mg/kg)	39,271

Copper (II) sulfate pentahydrate (E4)/copper	34,000
Iodine (mg/kg)	1,064
Anhydrous calcium iodate (E2)/iodine	1,000
Cobalt (mg/kg)	0,893
Cobalt (II) carbinate hydroxide (2:3) monohydrate	0,000
C18:2 g/kg	14,940
C18:3 g/kg	1,313
Vitamin A (E672)	25000,000
Vitamin D3 (E671)	3000,000
Vitamin E (All-rac-alpha-tocopheryl acetate)	150,000
Vitamin K3	4,000
Vitamin B1	20,000
Vitamin B2	25,000
Calcium-D-Pantothenate	30,000
Vitamin B6	10,000
Vitamin B9	4,000
Vitamin B12	64,000
Vitamin PP (Niacin)	60,000
Vitamin C	0,000
Biotin	304,000

Supplementary Table 2.
Significant correlations between *differences in FSIGTT MinMod parameters* and *changes in the relative abundance of bacterial genera in the gut microbiome* induced by training without aleurone supplementation. These bacterial families are significantly linearly correlated as assessed with the Spearman's correlation coefficient and they are listed per MinMod parameter (AIRg, SI, Sg and DI) from strongest to weakest correlation. Strong correlations are shown in bold

MinMod Parameters	Taxon lineage	Spearman correlation *r*	P value
**AIRg**	**k__Bacteria;p__Firmicutes;c__Clostridia;o__Clostridiales;f__Peptostreptococcaceae**	**0.732**	**0.003**
	k__Bacteria;p__Firmicutes;c__Erysipelotrichi;o__Erysipelotrichales;f__Erysipelotrichaceae	-0.590	0.023
	k__Bacteria;p__Firmicutes;c__Clostridia;o__Clostridiales;f__Veillonellaceae;g__Succinispira	-0.564	0.031
**SI**	**k__Bacteria;p__Proteobacteria;c__Gammaproteobacteria;__;__;__**	**0.718**	**0.004**
	k__Bacteria;p__Firmicutes;c__Bacilli;o__Lactobacillales;f__Streptococcaceae;g__Streptococcus	-0.664	0.009
	k__Bacteria;p__Firmicutes;c__Clostridia;o__Clostridiales;f__Peptostreptococcaceae;__	-0.661	0.009
	k__Bacteria;p__Bacteroidetes;c__Bacteroidia;o__Bacteroidales;f__Bacteroidaceae;g__BF311	0.596	0.021
	k__Bacteria;p__Firmicutes;__;__;__;__	-0.596	0.021
	k__Bacteria;p__Firmicutes;c__Clostridia;o__Clostridiales;__;__	-0.557	0.034
	k__Bacteria;p__Proteobacteria;c__Gammaproteobacteria;o__Pseudomonadales;f__Moraxellaceae;_	-0.555	0.032
	k__Bacteria;p__Cyanobacteria;c__4C0d-2;o__YS2;f__;g__	0.521	0.049
**Sg**	k__Bacteria;p__Proteobacteria;c__Betaproteobacteria;o__Burkholderiales;f__Oxalobacteraceae;__	-0.592	0.020
	k__Bacteria;p__Firmicutes;c__Clostridia;o__Clostridiales;f__Lachnospiraceae;g__Dorea	-0.556	0.032
**DI**	k__Bacteria;p__Firmicutes;c__Clostridia;o__Clostridiales;f__Lachnospiraceae;g__Clostridium	-0.654	0.010
	k__Bacteria;p__Firmicutes;c__Clostridia;o__Clostridiales;f__Veillonellaceae;g__Selenomonas	0,586	0.024
	k__Bacteria;p__Spirochaetes;c__Spirochaetes;o__Spirochaetales;f__Spirochaetaceae;g__Spirochaeta	0.570	0.026
	k__Bacteria;p__Bacteroidetes;c__Bacteroidia;o__Bacteroidales;f__Bacteroidaceae;__	-0.565	0.028
	k__Bacteria;p__Lentisphaerae;c__[Lentisphaeria];o__Victivallales;f__Victivallaceae;g__	0.528	0.043
